# Amyloid Precursor Protein (APP) Controls the Expression of the Transcriptional Activator Neuronal PAS Domain Protein 4 (NPAS4) and Synaptic GABA Release

**DOI:** 10.1523/ENEURO.0322-19.2020

**Published:** 2020-05-28

**Authors:** Rémi Opsomer, Sabrina Contino, Florian Perrin, Roberta Gualdani, Bernadette Tasiaux, Pierre Doyen, Maxime Vergouts, Céline Vrancx, Anna Doshina, Nathalie Pierrot, Jean-Noël Octave, Philippe Gailly, Serena Stanga, Pascal Kienlen-Campard

**Affiliations:** 1 CEllular and MOlecular Division-Alzheimer Dementia group, Institute of Neuroscience; 2 de Duve Institute, Ludwig Institute for Cancer Research; 3 CEllular and MOlecular Division-Laboratory of Neuropharmacology, Institute of Neuroscience; 4 CEllular and MOlecular Division-Laboratory of Cell Physiology, Institute of Neuroscience, Université Catholique de Louvain, B-1200, Brussels, Belgium

**Keywords:** amyloid precursor protein, CRISPR-Cas9, GABA, neuronal differentiation, NPAS4, transcriptome

## Abstract

The amyloid precursor protein (APP) has been extensively studied as the precursor of the β-amyloid (Aβ) peptide, the major component of the senile plaques found in the brain of Alzheimer’s disease (AD) patients. However, the function of APP per se in neuronal physiology remains to be fully elucidated. APP is expressed at high levels in the brain. It resembles a cell adhesion molecule or a membrane receptor, suggesting that its function relies on cell-cell interaction and/or activation of intracellular signaling pathways. In this respect, the APP intracellular domain (AICD) was reported to act as a transcriptional regulator. Here, we used a transcriptome-based approach to identify the genes transcriptionally regulated by APP in the rodent embryonic cortex and on maturation of primary cortical neurons. Surprisingly, the overall transcriptional changes were subtle, but a more detailed analysis pointed to genes clustered in neuronal-activity dependent pathways. In particular, we observed a decreased transcription of neuronal PAS domain protein 4 (NPAS4) in APP−/− neurons. NPAS4 is an inducible transcription factor (ITF) regulated by neuronal depolarization. The downregulation of NPAS4 co-occurs with an increased production of the inhibitory neurotransmitter GABA and a reduced expression of the GABA_A_ receptors α1. CRISPR-Cas-mediated silencing of NPAS4 in neurons led to similar observations. Patch-clamp investigation did not reveal any functional decrease of GABA_A_ receptors activity, but long-term potentiation (LTP) measurement supported an increased GABA component in synaptic transmission of APP−/− mice. Together, NPAS4 appears to be a downstream target involved in APP-dependent regulation of inhibitory synaptic transmission.

## Visual Abstract

**Figure:**
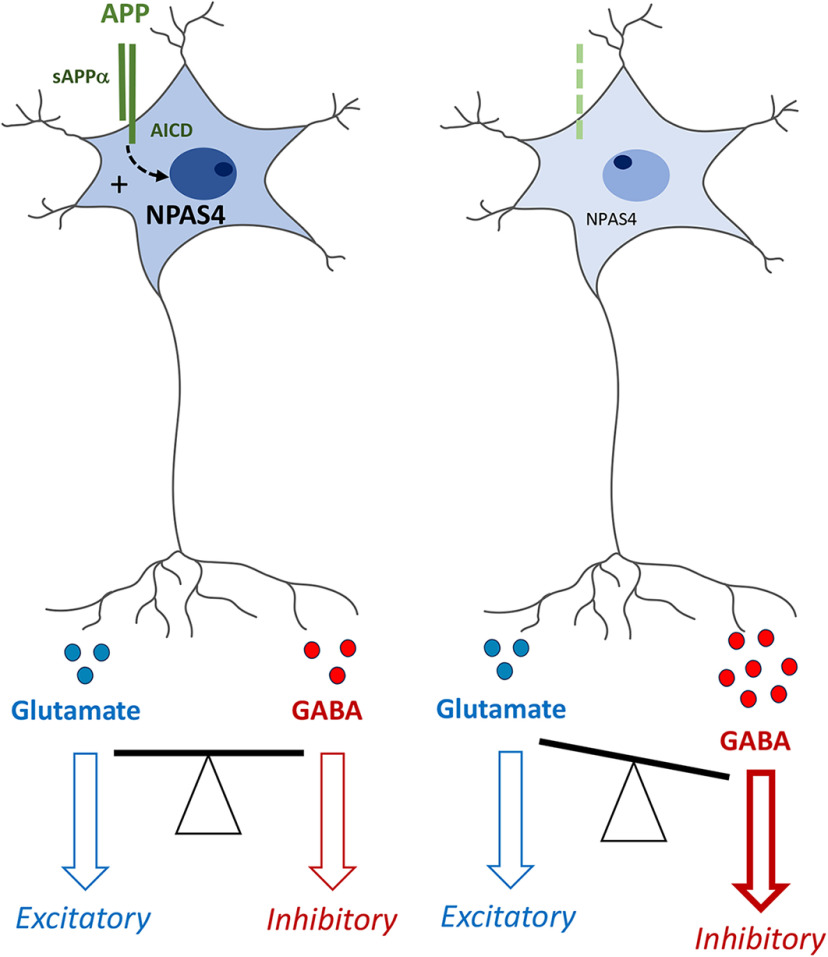

## Significance Statement

The amyloid precursor protein (APP) is a key player in Alzheimer**’**s disease (AD) pathogenesis. We report the downregulation of the activity-dependent transcription factor neuronal PAS domain protein 4 (NPAS4) in APP-deficient neurons, along with an increase in GABAergic neuron markers and GABA release, but not in excitatory glutamatergic markers. We identified NPAS4 as an APP target gene by a transcriptome analysis of APP+/+ versus APP−/− primary cortical neurons at different stages of differentiation. The downregulation of NPAS4 observed in APP−/− neurons was confirmed by APP silencing with a CRISPR/Cas9 approach. CRISPR/Cas9-silencing of NPAS4 mimicked APP deficiency and increased GABAergic markers. The activity-dependent transcription factor NPAS4 is therefore a key downstream target in the synaptic functions regulated by APP.

## Introduction

The physiological functions of amyloid precursor protein (APP) per se have been largely overlooked in comparison with its role of precursor of the β-amyloid (Aβ) peptide. Aβ deposition is a central event in Alzheimer**’**s disease (AD), but alterations of APP physiological functions are likely to play a key role in the pathogenesis.

APP belongs to the APP-like protein family (with APLP1 and APLP2, referred to as APLPs), expressed in most of the species. The APLP family results from several duplications and contraction events during evolution. The specific functions ascribed to each member are yet not clearly defined (for review, see Shariati and De Strooper, 2013). APP−/− mice show a subtle phenotype, with reduced body and brain weight, reduced locomotor activity, gliosis, mild axonal growth/white matter defects (Guo et al., 2012; Müller et al., 2012; Müller and Zheng, 2012). However, a large range of functions have been attributed to APP including neuronal proliferation, differentiation (Freude et al., 2011; Hu et al., 2013), and migration during embryogenesis (Young-Pearse et al., 2007). APP promotes neurite outgrowth (Hoe et al., 2009b), synapse formation and activity (Priller et al., 2006; Santos et al., 2009; Lee et al., 2010; Pierrot et al., 2013; Klevanski et al., 2015; Zou et al., 2016) in the CNS, or at the neuromuscular junction (Stanga et al., 2016). APP modulates the excitatory neurotransmission by interacting with AMPA (Lee et al., 2010) or NMDA receptors (Cousins et al., 2009; Hoe et al., 2009a). APP deficiency reduces paired pulse depression (PPD) in mice (Seabrook et al., 1999) and affects the expression of GABA receptors (Fitzjohn et al., 2000; Chen et al., 2017). Its overexpression induces hyperexcitability due to failure in GABAergic neurotransmission (Born et al., 2014), and triggers the GABA excitatory/inhibitory shift occurring during neuronal maturation (Doshina et al., 2017).

Tuning inhibitory/excitatory neurotransmission is instrumental in neuronal plasticity and memory formation. This process is regulated by a set of transcription factors known as inducible transcription factors (ITFs). ITFs belong to the immediate early genes (IEGs) family, and are sensitive to neuronal activity. They control the mechanisms that “reshape” synaptic inputs on neurons (West and Greenberg, 2011), and play a key role in neuronal plasticity and memory formation (Alberini, 2009; Loebrich and Nedivi, 2009; Leslie and Nedivi, 2011). Among them, neuronal PAS domain protein 4 (NPAS4) is robustly expressed on neuronal depolarization, and is involved in a transcriptional program that regulates neuronal firing responses to excitatory transmission by enhancing inhibition (Lin et al., 2008). Elevated activity of inhibitory neurons also induces NPAS4, promoting increased excitation onto the same neurons (Spiegel et al., 2014).

The molecular mechanisms underlying APP functions are still elusive, but several studies reported that APP function is mediated by the transcriptional regulation of so-called APP target genes, which is operated by the APP intracellular domain (AICD). An increasing list of AICD candidate genes has emerged from various models (for review, see Pardossi-Piquard and Checler, 2012). On the other hand, APP was also reported to regulate gene transcription independently of AICD release (Hicks et al., 2013; Pierrot et al., 2013). It is thus so far quite impossible to clearly define (1) the precise identity of APP target genes in neurons, (2) the related molecular pathways underlying APP-dependent gene transcription, and (3) how APP target genes relate to APP neuronal function.

In this study, we first aimed at identifying genes that are transcriptionally regulated by APP in primary neurons. We performed a transcriptome analysis (APP+/+ vs APP−/−) in primary cortical neurons at different stages of differentiation. Changes observed in global gene expression in the absence of APP were subtle. A more detailed pathway analysis indicated that expression of genes clustered in activity-dependent pathway, and among these the ITF NPAS4, were downregulated in the absence of APP after 7 d of culture. Strikingly, we observed that the amount of the inhibitory neurotransmitter GABA was increased in APP−/− neurons. This was supported by an increased expression of the glutamate decarboxylase 65 (GAD65) in the same context. Glutamate levels were not altered in APP−/− neurons. These observations were reproduced on acute silencing of APP by CRISPR-Cas9 editing. The knock-down of NPAS4 gave similar results. Neurophysiological investigations showed that EPSPs consecutive to a θ-burst stimulation (TBS) decreased in APP−/− supporting the increase release GABA, and suggesting increased inhibitory synaptic inputs in APP−/− neurons. Altogether, our data provide new insight into APP-dependent neuronal activity, indicating that NPAS4 is an APP downstream target gene, tuning the GABA-dependent activity in neuronal networks.

## Materials and Methods

### Antibodies, chemicals, and reagents

All media and reagents used for cell cultures were purchased from Thermo Fisher Scientific; fetal bovine serum (FBS) was purchased from Biowest. Analytical grade solvents and salts were purchased from Sigma-Aldrich. sAPPα (S9564) and DAPI (D9542) were from Sigma-Aldrich, Triton X-100 from Merck, and TriPure Isolation Reagent from Roche. Microarray analysis kits were from Affymetrix. All reagents for RNA processing or cDNA synthesis were from Bio-Rad, and primers were from Sigma-Aldrich. BCA Protein Assay kit was from Thermo Fisher Scientific. NuPAGE reagents were from Invitrogen. Polyvinylidene fluoride (PVDF) and nitrocellulose membranes were from Merck Millipore or GE Healthcare. Nonfat dry milk was from Merck. Western Lighting Plus-ECL reagents were from PerkinElmer and Fluoprep mounting medium from bioMérieux. Lentivirus were prepared with Acrodisc 0.45-µm filters (Pall) and LentiX Concentrator reagent (Clontech).

The following antibodies were used: APP NT 22C11 (catalog #MAB348, Merck Millipore), anti-human APP W0-2 (catalog #MABN10, Merck Millipore), anti-APP CT Y188 (catalog #ab32136 Abcam), anti-APLP1 (catalog #171615, Calbiochem EMD Biosciences–Merck), anti-APLP2 (catalog #171616, Calbiochem EMD Biosciences–Merck), anti-GAPDH 14C10 (catalog #2118, Cell Signaling), anti-microtubule associated protein 2 (MAP2; catalog #M4403, Sigma-Aldrich), anti-GAD65 (D5G2, catalog #5843 Cell Signaling), anti-mouse IgG, HRP whole antibody anti-rabbit IgG HRP (catalog #NA931-1ML, GE Healthcare), whole goat anti-mouse antibody (catalog #NA934-1ML, GE Healthcare) Alexa Fluor 488, goat anti-mouse; Alexa Fluor 568, goat anti-rabbit; Alexa Fluor 647 and DAPI were purchased from Thermo Fisher Scientific. Glutamate assay kit was from Abcam and GABA ELISA from Cloud-Clone Corporation; 70-µm Falcon Cell Stainers were from Thermo Fisher Scientific.

### Animal models

All animal procedures were performed in accordance with the regulations and policies of the University animal care committee. APP+/+ and APP−/− mice were obtained from The Jackson Laboratory as C57Bl6/J and backcrossed for more than six generations in CD1 genetic background. Animals were housed on a 12/12 h light/dark cycle in standard animal care facility with access to food and water *ad libitum*. Heterozygous animals (APP+/−) were bred and crossed to obtain embryos of either sex from the three different genotypes (APP+/+, APP+/−, and APP−/−) in the same litter.

### Primary culture and treatments

Primary cultures of cortical neurons were prepared from E18 mouse embryos. Cortices were dissected and dissociated in HBSS without calcium and magnesium and the mixture was centrifuged on FBS for 10 min at 1000 × *g* to pellet cells. Cells were plated at 200,000 cells/cm^2^ in culture dishes pre-treated with 10 µg/ml of poly-L-lysine in PBS and cultured (37°C, 5% CO_2_, and humidified atmosphere). Cells were cultured for 3–14 d *in vitro* (DIV) in Neurobasal medium enriched with 2% v/v B-27 supplement medium and 1 mm L-glutamine. Half of the medium was renewed every 2–3 d. Treatments with 20 nm of soluble APPα (sAPPα) were performed for 16 h after 6 d of culture (DIV6).

For primary cultures of astrocytes, cortices from pups were collected at postnatal day 2 and mechanically dissociated. Astrocytes were isolated using a 30% Percoll gradient and seeded into gelatin-coated tissue culture flasks. Cells were left to proliferate for 14 d at 37°C–5% CO_2_ in DMEM-glutaMAX medium supplemented with 10% FBS, 50 mg/ml penicillin–streptomycin, and 50 mg/ml fungizone. Medium was renewed after 7 d, cells were passaged after 14 d and further cultured in DMEM-glutaMAX with 10% FBS. Two days after passage, FBS was reduced to 3%, and medium was supplemented with the growth factor cocktail G5. All experiments/treatments were performed 7 d after, referred to as DIV7 for astrocytes. For NPAS4-induction analysis, neurons and astrocytes at DIV7 were depolarized with 50 mm KCl for 2–4 h.

### RNA extraction, transcriptome analysis, and qRT-PCR

Total RNA was extracted by TriPure Isolation reagent according to the manufacturer’s protocol. RNA samples were suspended in DEPC-treated water and RNA concentration was measured (OD 260 nm) on BioSpec-nano spectrophotometer (Shimadzu Biotech). For microarray analysis, RNA quality was monitored by capillary electrophoresis using the Agilent 2100 Bioanalyzer instrument with the Agilent RNA 6000 Nano kit (Agilent); 250 ng of total RNA per sample was amplified and labeled using GeneChip WT PLUS Reagent kit (Affymetrix) before hybridization over night at 45°C on GeneChip Mouse Transcriptome 1.0 Array. The chip was washed on the GeneChip Fluidics Station 450 followed by scanning on a GeneChip Scanner on Affymetrix microarray platform. For quantitative PCR, RNA samples were reversed transcribed using iScript cDNA Synthesis kit and real-time PCR was performed in an iCycler MyIQ2 multicolor-real-time PCR detection system using iQ SYBR Green supermix kit (Bio-Rad). A standard curve was established for relative quantification with a fourfold dilution series (from 100 to 0.0097 ng) of a cDNA template mix. Relative quantification was calculated by the 2^ΔΔCT^ method (*Gapdh* as housekeeping control) and then normalized (percentage or fold) to the control condition (Ct). Primer used (forward/reverse) are:

*Gapdh*5′-ACCCAGAAGACTGTGGATGG-3′/5′- ACACATTGGGGGTAGGAACA-3′;

*Npas4*5′-GCTATACTCAGAAGGTCCAGAAGGC-3′/5′-TCAGAGAATGAGGGTAGCACAGC-3′;

*Egr1*5′-TCCTCTCCATCACATGCCTG-3′/5′-CACTCTGACACATGCTCCAG-3′;

*Egr3*5′-GACTCGGTAGCCCATTACAATC-3′/5′-ACTTTCCCAAGTAGGTCACGG-3′.

### Western blotting

Cells were solubilized and sonicated in lysis buffer (20% glycerol, 4% SDS, 125 mm Tris-HCl; pH 6.8) containing a cocktail of proteases and phosphatases inhibitors (Roche, Basel, Switzerland). When performed on tissue extracts, mice were euthanized (ketamine/xylazine injection) and brains were dissected after perfusion with ice-cold sterile PBS. Cortices and hippocampi were isolated and quickly frozen in liquid nitrogen. Tissues were crushed using mortar pestle method. For brain protein extraction, samples were homogenized in RIPA buffer [1% (w/v) NP40, 0.5% (w/v) deoxycholic acid, 0.1% (w/v) SDS, 150 mm NaCl, 1 mm EDTA, and 50 mm Tris; pH 7.4] containing proteases and phosphatases inhibitors cocktail. The samples were clarified by centrifugation at 20,000 × *g*. Protein concentrations were determined with a BCA kit. Samples were prepared with NuPAGE LDS sample buffer (4×) and 50 mm DTT and then heated for 10 min at 70°C; 10–40 µg of proteins or 22 µl of culture medium were loaded per well for migration followed by transfer onto PVDF or nitrocellulose membranes. For APP C-terminal fragments, proteins were transferred on nitrocellulose (0.1 µm). Membranes were blocked in nonfat dry milk (5% in PBS, 0.05% Tween 20) and immunoblotted with anti-APP NT (22C11, 1:500), anti-APP CT (Y188, 1:500), anti-APLP1 (1:1000), anti-APLP2 (1:1000), and anti-GAPDH (1:25,000). Blots were revealed using ECL and signal quantification was performed using GelQuant.NET software (http://www.BiochemLabSolutions.com).

### Immunocytofluorescence (ICF)

Neurons grown at 100,000 cells/cm^2^ per well on poly-L-lysine coated coverslips were fixed for 15 min in PBS/4% paraformaldehyde and washed twice in PBS for 5 min. Permeabilization and blocking steps were done in PBS/5% skimmed milk/0.3% Triton X-100. Antibodies were incubated in PBS/5% skimmed milk/0.1% Triton X-100 (M1TPBS). Primary antibodies dilutions used were as following: mouse anti-MAP2 (1:1000), rabbit anti-APP (Y188, 1:100), and rabbit anti-GAD65 (D5G2, 1:100). Secondary antibodies dilutions used were as following: goat anti-mouse Alexa Fluor 488 (1:500), goat anti-mouse Alexa Fluor 568 (1:500), and goat anti-rabbit Alexa Fluor 647 (1:500). Images were acquired on Evos FL Auto microscope (Invitrogen) with GFP (Alexa Fluor 488 or native GFP), TxRed (Alexa Fluor 568), and CY5 (Alexa Fluor 647) EVOS LED light cubes and analyzed with ImageJ software. For the quantification of signal area, 10× or 20× magnification images were identically thresholded for APP+/+ and APP−/−, or Ct and CRISPR-*NPAS4*. The area of thresholded images was measured and normalized to the number of cells counted by DAPI staining. For the quantification of the APP expression intensity, image acquisition was performed using 40× objective coverslip-corrected (Thermo Fischer Scientific, AMEP4699) in GFP, CY5 (APP) and DAPI channels. A total of 12, 19, and 19 images were acquired and processed to obtain 33, 46, and 51 neurons in the analysis, respectively for CRISPR control (Ct), Oligo2, and Oligo17. GFP channel images were first eight-bit transformed and thresholded to highlight only GFP staining. A region of interest (ROI) was delimited around GFP+ neurons in the GFP channel (green) using “wand tool” in ImageJ software and transposed to CY5 (APP) channel (blue). ROI mean intensity is measured with the Analyze tool of ImageJ software.

### AICD and CRISPR/Cas9 lentiviral constructions, production, and viral transduction

Lentiviruses were used to express AICD in neurons. AICD50 tagged at the c-terminal part with hemagglutinin (HA) was cloned into pLenti CMV/TO Puro lentiviral vector (Addgene reference #17482). pLenti CMV/TO Puro empty served as a control (Ct). A lentiviral vector-based approach was also used to deliver the CRISPR-Cas9 system. sgRNAs Oligo2 and Oligo17 were designed using on/off-target score algorithm to target the *APP* mouse gene (gene ID: 11820), and sgRNA CRISPR-*NPAS4* to target the *NPAS4* mouse gene (gene ID: 225872). sgRNAs were cloned in a lentiviral vector delivering sgRNA, SpCas9 and coexpressing eGFP (Addgene #57818) according to author instructions (Heckl et al., 2014). The negative Ct used was the lentiviral construct without sgRNA but expressing SpCas9 and eGFP. sgRNA used are (sequence/PAM/specificity score):

*APP Oligo2*5′-GTGGAAGATCCGCCGCGCCC-3′/TGG/95;

*APP Oligo17*5′-GTACCCACTGATGGCAACGC-3′/CGG/92;

*Npas4*5′-GACCCTTGCGAGTGTAGATGC-3/AGG/83.

All lentiviral vectors were validated by sequencing (Beckman Coulter Genomics) before production and purification using the Plasmid Midi kit (QIAGEN). Production was conducted by transfecting HEK293-T cells in 10-cm dishes (2 × 106 cells/dish) with lentiviral CRISPR-Cas9 vectors, pCMV-dR8.2 (Addgene #12263), and pMD2.G (Addgene #12259). After 48 h, the supernatant was filtered and incubated with 1/3 (v/v) of LentiX Concentrator for 90 min on ice. The collected supernatant was centrifuged at 1500 × *g* for 45 min at 4°C, the pellet was resuspended in 20 µl per dish of Neurobasal Medium and stored at −80°C until use.

Neurons were infected with CRISPR-Cas9 lentiviruses 1 d after plating (DIV1). Typically, 20 µl of concentrated virus were used to infect 800,000 cells per well in a 12-well culture dish. The medium was completely changed after 24 h, and a half-media change was performed every 2–3 d thereafter. The neurons were harvested at DIV7 or as indicated.

### Toxicity assay

Cell viability on lentiviral infection was measured by lactate dehydrogenase (LDH) release in the culture medium using Cytotoxicity Detection kit (Sigma-Aldrich), according to the manufacturer’s instructions. Relative absorbance was measured at 490 nm using a VICTOR Multilabel Plate Reader (PerkinElmer). Background LDH release was determined in non-infected control cultures.

### Flow cytometry and cell sorting

At DIV7, infected neurons were briefly rinsed with PBS and trypsinized for 2 min. Neurons were mechanically dissociated and filtered through 70-µm Falcon Cell Strainers in 50-ml tube containing FBS. Cells were pelleted by centrifugation at 1000 × *g* for 5 min and resuspended in PBS/1% FBS/1 mm EDTA. TO-PRO-3 iodide (Thermo Fisher Scientific) was used to stain dead cells and exclude them for the sorting. Cells were sorted using a BD FACSAriaIII cell sorter (BD Biosciences). The sort parameters used were the following: nozzle 100 µm, sheath pressure 20 psi, drop frequency 30 kHz, and sort precision 16-32-0. Sample and collection tubes were maintained at 4°C throughout the procedure. GFP-negative and positive cells were harvested in PBS/1% FBS/1 mm EDTA, centrifuged at 12,000 × *g* for 2 min and homogenized in TriPure Isolation reagent for RNA extraction.

### Glutamate and GABA measurements

Neurons were grown at 200,000 cells/cm^2^ in 12-well culture dish. Glutamate and GABA were measured in cells lysates and culture media at DIV7. Media were harvested, centrifuged to pellet and remove cellular debris, treated with a cocktail of proteases inhibitors and frozen at −20°C until use. Cells were scrapped in ice-cold PBS, pelleted by centrifugation (12,000 × *g* for 3 min at 4°C), quickly frozen in liquid nitrogen and kept at −80°C until use. Glutamate and GABA assays were performed according to the manufacturer’s protocol (Abcam). For both, cells were lysed by five cycles of thawing and freezing in PBS and centrifuged at 12,000 × *g* for 10 min at 4°C. Supernatants were used for the quantification and normalized on protein content. Media were directly used for quantification.

### Calcium imaging

Changes in intracellular [Ca^2+^] were measured in single neurons using the calcium sensitive fluorescent dye fura-2 (Invitrogen). Neurons were grown on poly-L-lysine-coated 15 mm diameter coverslips and were loaded with 2 μm fura-2 AM for 40 min in a Krebs buffer (10 mm HEPES, 135 mm NaCl, 6 mm KCl, 2 mm CaCl_2_, 1.2 mm MgCl_2_, and 10 mm glucose; pH 7.4) at room temperature. Coverslips were rinsed once and then mounted on a heated (37°C) and perfused microscope chamber (Warner Instrument Corporation). While continuously perfused with heated Krebs buffer, fura-2-loaded neurons were excited successively at 340 and 380 nm (excitation light was obtained from a xenon lamp coupled to a monochromator) for 2 × 100 ms. Emitted fluorescence was monitored at 510 nm using a charged coupled device sensor camera coupled to an inverted Olympus IX70 microscope (TILL photonics). Fluorescence intensities from each single neuron was recorded separately, corrected for the background and combined (fluorescence ratio F340/F380) using the software TILLvisION version 3.3. Calcium signals were measured on application (perfusion) of 30 μm glutamate in Krebs buffer. A total of 70–80 cells were analyzed in each experiment (coverslips) and non-neuronal cells were excluded from analysis as previously described by Pickering et al. (2008). Changes in intracellular [Ca^2+^] were calculated from fluorescence emission intensity ratios (F340/F380). These changes were expressed as normalized fluorescence where every measurement of F340/F380 was divided by the basal fluorescence value corresponding to the mean of signals measured during a period of 25 s in basal condition (before the glutamate stimulation). To estimate the amplitude of the response to glutamate, the area under curve (AUC) was calculated using GraphPad Prism (GraphPad Software).

### Field potential recordings

Three-month-old mouse brain slices were prepared as described in (Lepannetier et al., 2018). EPSPs were evoked through a bipolar stimulating electrode placed in the Schaffer collaterals (SC) and recorded by the AxoClamp 2B (Molecular Devices) amplifier through a glass electrode placed in the CA1 region (stratum radiatum). Stimuli consisted of 100 µs pulses of constant currents with intensity adjusted to produce 35% of the maximum response every min. Responses were digitized by Digidata1322A (Molecular Devices) and recorded to a computer using WinLTP software (Anderson and Collingridge, 2007). Long-term potentiation (LTP) was induced by applying a TBS consisting in four trains of five pulses (100 Hz) separated by a 200-ms interval. Slopes of field EPSPs (fEPSPs) responses were expressed normalized to the pretreatment baseline, defined as 100%.

### Electrophysiology of cultured neurons

Patch-clamp recordings of primary neurons at DIV7 were conducted at room temperature, using an EPC-9 amplifier controlled by PatchMaster software (HEKA Elektronik). GABA was applied by pressure ejection using a Picospritzer. The patch pipettes were pulled with a resistance of 4–7 MΩ using a DMZ-Universal Puller (Zeitz Instruments). Series resistances were compensated (75–90%) and periodically monitored. The following extracellular solution was used: 140 mm NaCl, 5 mm KCl, 1 mm CaCl_2_, 1 mm MgCl_2_, 10 mm glucose, and 10 mm HEPES; pH 7.4. The pipette solution had the following composition: 140 mm CsCl, 10 mm EGTA, 0.3 mm Mg2ATP, 0.3 mm CaCl_2_, and 10 mm HEPES; pH 7.25. To prevent network activity, the experiments were performed in the presence of 1 μm tetrodotoxin (TTX), 10 μm 6-cyano-7-nitroquinoxaline-2,3-dione disodium (CNQX), 20 μm D-(-)-2-amino-5-phosphonopentanoic acid (D-AP5), and 100 nm CGP55845.

### Statistical analysis

For microarray analysis, raw data were analyzed using Bioconductor (R environment). Robust multiarray average (RMA) was used for background correction, normalization, probe level intensity calculation and probe set summarization. Gene expression values were compared between APP+/+ and APP−/− neurons at different stage of development DIV3, DIV7, and E18 using the R-Limma (Linear Models for MicroArray Data) package. Benjamini–Hochberg procedure was used for multiple testing corrections. Only transcripts with an Entrez ID were kept among the raw data to facilitate the analysis. Gene set enrichment analysis was performed on differentially expressed genes sets after the ROAST (rotation gene set tests for complex microarray experiments; Wu et al., 2010) procedure to identify KEGG pathways modified in absence of APP for all conditions (E18, DIV3, and DIV7). The data obtained have been deposited in NCBI’s Gene Expression Omnibus (Edgar et al., 2002) and are accessible through GEO Series accession number GSE112847. Otherwise, statistical analyses were performed using GraphPad Prism (GraphPad Software). Gaussian distribution was assessed by Kolmogorov–Smirnov test (GraphPad Prism). Parametric test was applied if the data followed normal distribution. Otherwise non-parametric tests were used. When two groups were compared, parametric Student’s *t* test or non-parametric Mann–Whitney test were used. When more than two groups were compared, parametric ANOVA with indicated *post hoc* tests or non-parametric Kruskal–Wallis were used. Significance is indicated as ns = non-significant; *p < 0.05, **p < 0.01, ***p < 0.001. The number of samples per condition in one experiment (*n*) and the number of biological replicates (*N*) are indicated in figure legends.

## Results

### APP-dependent expression of NPAS4 in differentiated primary neuron cultures

Transcriptome analysis were performed on primary neurons and embryonic cortex according to the workflow described in Extended Data Figure 1-1*A*. Neurons from embryonic cortex (E18) were cultured for 3 or 7 DIV (DIV3 or DIV7) and up to DIV14 when necessary. Characterization of APP protein family expression indicated an increase in APP, APLP1, and APLP2 on differentiation with a peak of expression at DIV7–DIV8 (Extended Data Fig. 1-1*B*,*C*), supporting an important role of APP protein family in neuronal maturation. APLP1 and APLP2 levels were similar in APP−/− neurons and APP+/+ at any time point of differentiation (Extended Data Fig. 1-1*D*). Thus, the results obtained here in APP−/− neurons are related to the loss of APP and not to indirect effects resulting from upregulation or downregulation of APLP1 or APLP2 in the absence of APP.

**Figure 1.**
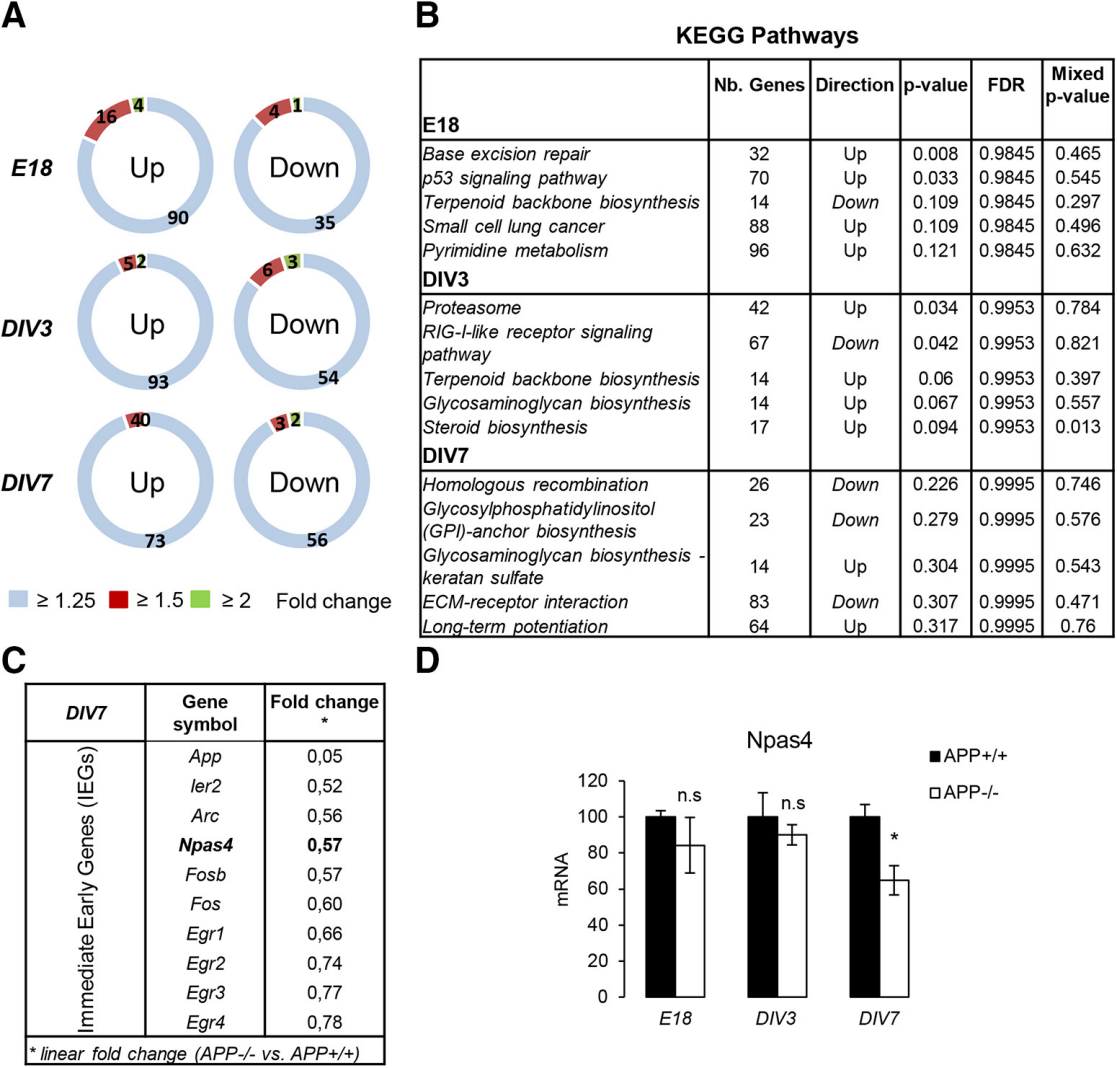
APP-dependent expression of NPAS4 in young differentiating neuronal culture. Summary of transcriptome analysis performed with the GeneChip Mouse Transcriptome Array 1.0 (Affymetrix). The characterization of the model and the experimental workflow are described in Extended Data Figure 1-1. Data were processed in triplicate (*N* = 3) for each experimental time point (E18, DIV3, DIV7). Non-coding transcripts and alternative splicing products were detected by these arrays, but only transcripts of coding transcripts have been considered here. For all the transcripts, adjusted *p* > 0.05 except for APP (internal control, *p* < 0.05). ***A***, Number of upregulated and downregulated coding transcript in APP−/− versus APP+/+ primary neurons at E18, DIV3, and DIV7. Linear fold changes have been set at 1.25, 1.5, and 2. ***B***, KEGG pathway analysis (http://www.genome.jp/kegg/pathway.html) at E18, DIV3, DIV7 (APP−/− vs APP+/+) to identify networks molecular pathways (or interaction networks) in which differentially expressed genes are clustered. The five most modified pathways are displayed for each time point, with the number of genes potentially lated or downregulated. ***C***, IEGs expression in APP−/− versus APP+/+ primary neurons at DIV7 and their respective fold change (APP−/− vs APP+/+) in microarray analysis at DIV7. ***D***, Neuronal PAS 4 domain (*NPAS4*) mRNA level was measured by qPCR at E18, DIV3, and DIV7 (*n* = 6, *N* = 3). Results (mean ± SEM) are expressed as percentage of controls (APP+/+); n.s. = non-significant, *p = 0.0242, Student’s *t* test. mRNA levels of two other IEGs (Egr1 and Egr3) were measured in the same conditions (Extended Data Figure 1-2).

Previous studies indicated that APP-dependent gene transcription involves the release of its intracellular domain or AICD. AICD is detectable in the nucleus of primary neurons at DIV6–DIV7 (Kimberly et al., 2005), suggesting that AICD-dependent gene transcription should be temporally restricted. We monitored AICD production at DIV7 in APP+/+, APP+/−, and APP−/− cultures (Extended Data Fig. 1-1*E*). AICD was only readily detectable in APP+/+ neurons at a high exposure time, confirming (1) that it is a transient peptide (Huysseune et al., 2007) with a restricted temporally expression pattern in primary neurons and (2) that APP-dependent transcriptional regulation may occur at a defined time period (around DIV7). We thus performed microarray experiments at several differentiation stages (summarized in Extended Data Fig. 1-1*A*) to track genome-wide expression changes in APP +/+ and APP−/− primary cortical neurons at DIV3 (immature neuronal network, no AICD), DIV7 (neuronal network with detectable AICD) and in E18 cortical tissue (embryonic tissue). We used Affymetrix GeneChip Mouse Transcriptome 1.0 Array and performed data analysis with the R-Limma package (Ritchie et al., 2015). The chips used allowed profiling of the expression of coding and non-coding genes (lncRNA, miRNA, pseudogene…) as well as alternative splicing events. Transcriptome analysis was performed in triplicate (*N* = 3 independent cultures) for each condition (E18, DIV3, and DIV7). We focused on differentially expressed coding genes, although data were also collected for non-coding RNAs. Strikingly, the overall changes observed (fold changes) were moderate in all conditions (E18, DIV3, and DIV7). Few coding transcripts appear to be differentially expressed when the specific fold change (linear) is set at 1.25, 1.5, or 2 (Fig. 1*A*). The Benjamini–Hochberg multiple correction test did not reveal any robust differential gene expression (adjusted *p* < 0.05) excepted for *APP* (positive control). To note, we did not measure significant change in the expression of genes previously identified as AICD target genes (Pardossi-Piquard and Checler, 2012). Gene enrichment analysis was further performed using the ROAST (rotation gene set test for complex microarray experiment) procedure to identify a molecular interaction/reaction networks diagram known as the KEGG pathway (Kanehisa and Goto, 2000) altered in the absence of APP. The first five pathways (in terms of significance), the number of genes modified as well as their direction are shown in Figure 1*B*. For instance, extracellular matrix (ECM)-receptor interaction and LTP pathways appeared to be modulated in absence of APP at DIV7. APP shares the structure of transmembrane receptors and cell adhesion proteins that activate cell-ECM pathways. LTP is a major mechanism in memory formation and learning. Both of these pathways have been associated to APP function (Cáceres and Brandan, 1997; Seabrook et al., 1999; Puzzo et al., 2011). We kept this pathways analysis to further investigate the regulation of candidate genes relevant to APP functions. In a set of arrays from a primary neuron at DIV7 (APP+/+ vs APP−/−), we noticed a downregulation of ITFs or IEGs in APP−/− neurons (Fig. 1*C*). Among them, the activity-dependent transcription factor NPAS4. NPAS4 is a neuron-specific ITF, known to be regulated by neuronal depolarization. We confirmed by qPCR that the NPAS4 mRNA level was decreased at DIV7 in APP−/− neurons, but neither at DIV3 nor in the cortex at E18 (Fig. 1*D*). To note, the expression of other IEGs (*Egr1* and *Egr3*) previously reported to be APP downstream targets (Hendrickx et al., 2013, 2014) was not altered in our experiments (Extended Data Fig. 1-2).

10.1523/ENEURO.0322-19.2020.f1-1 Extended Data Figure 1-1 Experimental workflow and model characterization. ***A***, Experimental design used for the study (E18); neurons were cultured and experiments were mainly carried out after 3 and 7 DIV (DIV3 and DIV7). Transcriptome analysis was performed on embryonic cortex (E18) and at DIV3 or DIV7. ***B***, APP, APLP1, and APLP2 expressions were analyzed by Western blotting at the indicated days of culture in APP+/+ neurons. ***C***, Quantification of APP, APLP1, and APLP2 protein expression over time in APP+/+ neurons. Accumulation is represented as fold change over the signal measured at day 0. Quantification was performed from one neuronal culture. ***D***, APLP1 and APLP2 expressions are not modified in cortical tissue at E18 and primary neuron cultures at DIV3 and DIV7 in absence of APP. Expression of APP, APLP1, APLP2 was analyzed by Western blotting of cells lysates from APP+/+ and APP−/− primary neuron cultures. ***E***, Samples from primary cultures at DIV7 (APP+/+, APP+/−, and APP−/− neurons) were probed (Western blotting) with an antibody directed against APP C terminus for APP C-terminal fragments (CTFs) and AICD. Low and high exposures of a typical blot are shown. Arrows indicate the expected position of APP holoprotein, APP CTFs, and AICD. Download Figure 1-1, TIF file.

**Figure 2.**
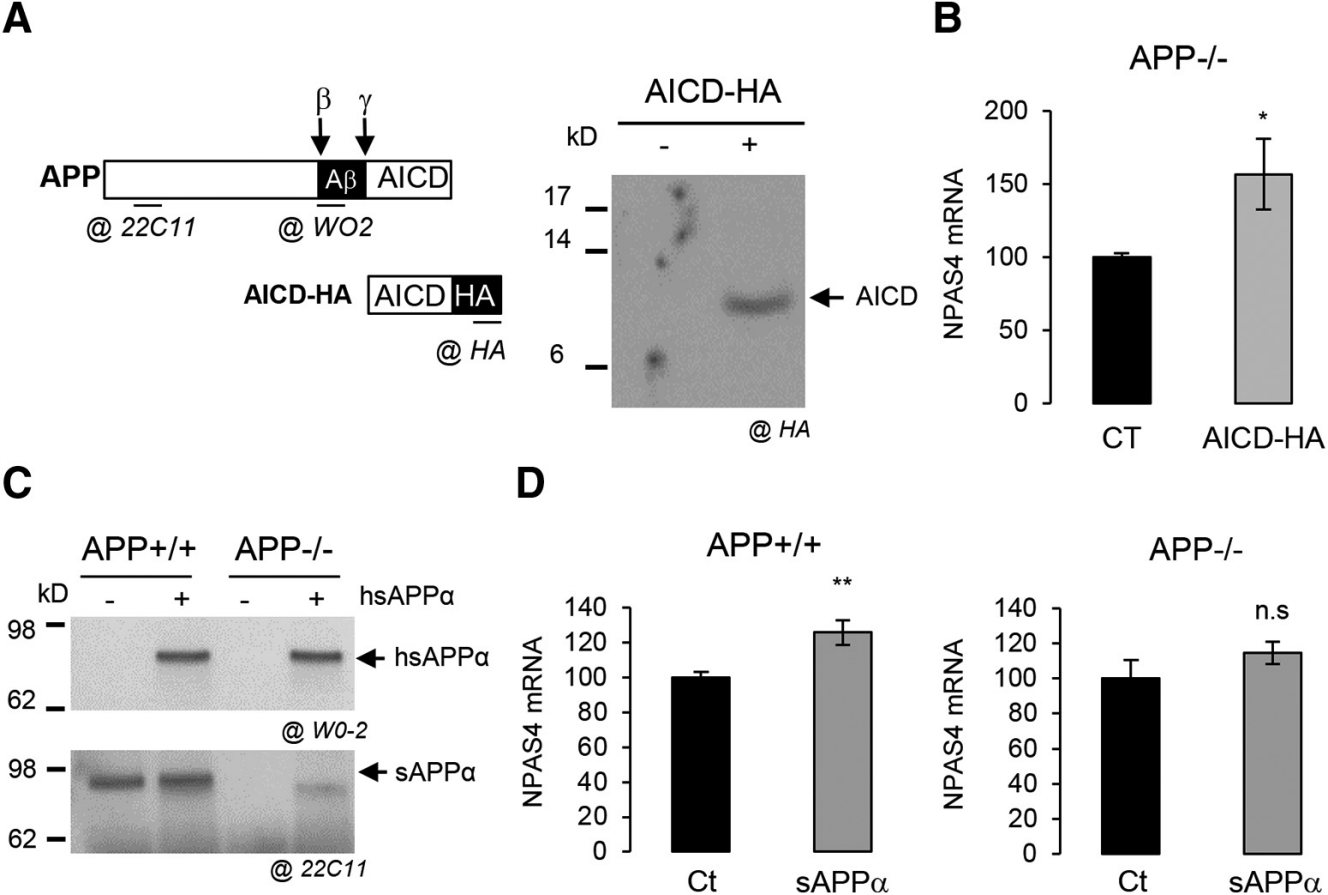
APP metabolites regulate NPAS4 expression. ***A***, Schematic representation of APP, its fragments, the AICD-HA construct along with and the localization of the epitopes recognized by the different antibodies used. Western blot analysis of AICD-HA expression after 3 d of lentiviral infection in cells with control or AICD-HA-expressing vectors. Total cell lysate was analyzed with anti-HA antibody. ***B***, Quantification by qPCR of NPAS4 mRNA in neurons APP−/− at DIV7 infected with lentiviral vector expressing AICD-HA (*n* = 6, *N* = 3). Results are expressed as percentage of control (Ct; mean± SEM); *p = 0.0291, Student’s *t* test. ***C***, Medium of sAPPα-treated APP+/+ or APP−/− neurons was subjected to Western blot analysis using anti-human APP antibody (clone W0-2) to detect the exogenous human sAPPα (h sAPPα) and an anti-mouse APP antibody (clone 22C11) to detect both endogenous and exogenous sAPPα (h + m sAPPα). Medium was collected after 16 h of treatment. ***D***, Quantification by qPCR of NPAS4 mRNA level in APP+/+ (*n* = 8, *N* = 4) or in APP−/− neurons at DIV7 treated with 20 nm sAPPα for 16 h (*n* = 6, *N* = 3). Results are expressed as percentage of control (Ct; mean ± SEM); **p = 0.0055, n.s. = non-significant, Student’s *t* test. Given that primary cultures of cortical neurons at DIV7 also contain astrocytes, the astrocytic pattern of NPAS4 expression is described in Extended Data Figure 2-1.

10.1523/ENEURO.0322-19.2020.f1-2 Extended Data Figure 1-2 Expression of Egr1 and Egr3 is not modified in APP−/− neurons. Egr1 and Egr3 expressions were evaluated in APP+/+ versus APP−/− primary neurons at DIV7. Egr1 mRNA (***A***) and Egr3 mRNA (***B***) levels was measured by qPCR (*n* = 6, *N* = 3) at DIV7. Results (mean ± SEM) are given as percentage of controls (APP+/+); n.s. = non-significant, Student’s *t* test. Download Figure 1-2, TIF file.

### Control of NPAS4 expression by APP

Since AICD, produced at DIV7, is reported to mediate APP nuclear signaling (Belyaev et al., 2010), we analyzed its involvement in NPAS4 expression. We transduced primary neurons with a lentiviral vector expressing the five C-terminal amino acids of APP (AICD) fused at the C terminus to the HA tag. AICD-HA is detectable in infected cells (Fig. 2*A*) and AICD expression in APP−/− neurons significantly increased NPAS4 mRNA levels (Fig. 2*B*), indicating that AICD is involved in the transcriptional regulation of NPAS4. As some of the APP functions were found to rely on its extracellular soluble fragment (sAPPα), we tested whether the sAPPα can regulate NPAS4 expression per se Treatment of neuronal cultures with 20 nm human sAPPα (Fig. 2*C*) significantly increased NPAS4 mRNA levels in APP+/+ neurons, but not in APP−/− neurons (Fig. 2*D*). Together, these data indicate that (1) AICD is likely to be involved in APP-dependent NPAS4 transcription, (2) sAPPα triggers NPAS4 expression, but only in a context where endogenous APP is expressed. Importantly, glial cells (∼16% of total cells in primary cultures) could indirectly contribute to these observations. We found that the absence of APP did not change the astrocytic pattern of primary cultures, and that astrocytes did not readily express NPAS4 in contrast to neuron (Extended Data Fig. 2-1). Moreover, NPAS4 is strongly induced as expected by depolarization only in neurons (Sun and Lin, 2016). Together, this indicated that NPAS4 is a downstream transcriptional target that could be involved in APP neuronal functions.

10.1523/ENEURO.0322-19.2020.f2-1 Extended Data Figure 2-1 Astrocytes in primary neuron culture and their implication in Npas4 expression. ***A***, Primary culture of cortical neurons at DIV7. Cultures were immunostained with the neuron-specific protein MAP2 (green), the glial-specific protein GFAP (red) and the DAPI (light blue). Scale bar = 400 µm. ***B***, Quantification of neurons (MAP2+) and astrocytes (GFAP+) in the primary cortical culture. At least five fields per coverslip were analyzed for APP+/+ and APP−/− cultures in two independent experiments (*n* ≥ 5, *N* = 2). Results are expressed as the ratio of MAP2+ (neurons) and GFAP+ (astrocytes; mean ± SEM); n.s. = non-significant, Mann–Whitney test. ***C***, Western blot analysis of NPAS4 induction in neurons and astrocytes after depolarization with 50 mm KCl for 2 h. Download Figure 2-1, TIF file.

**Figure 3.**
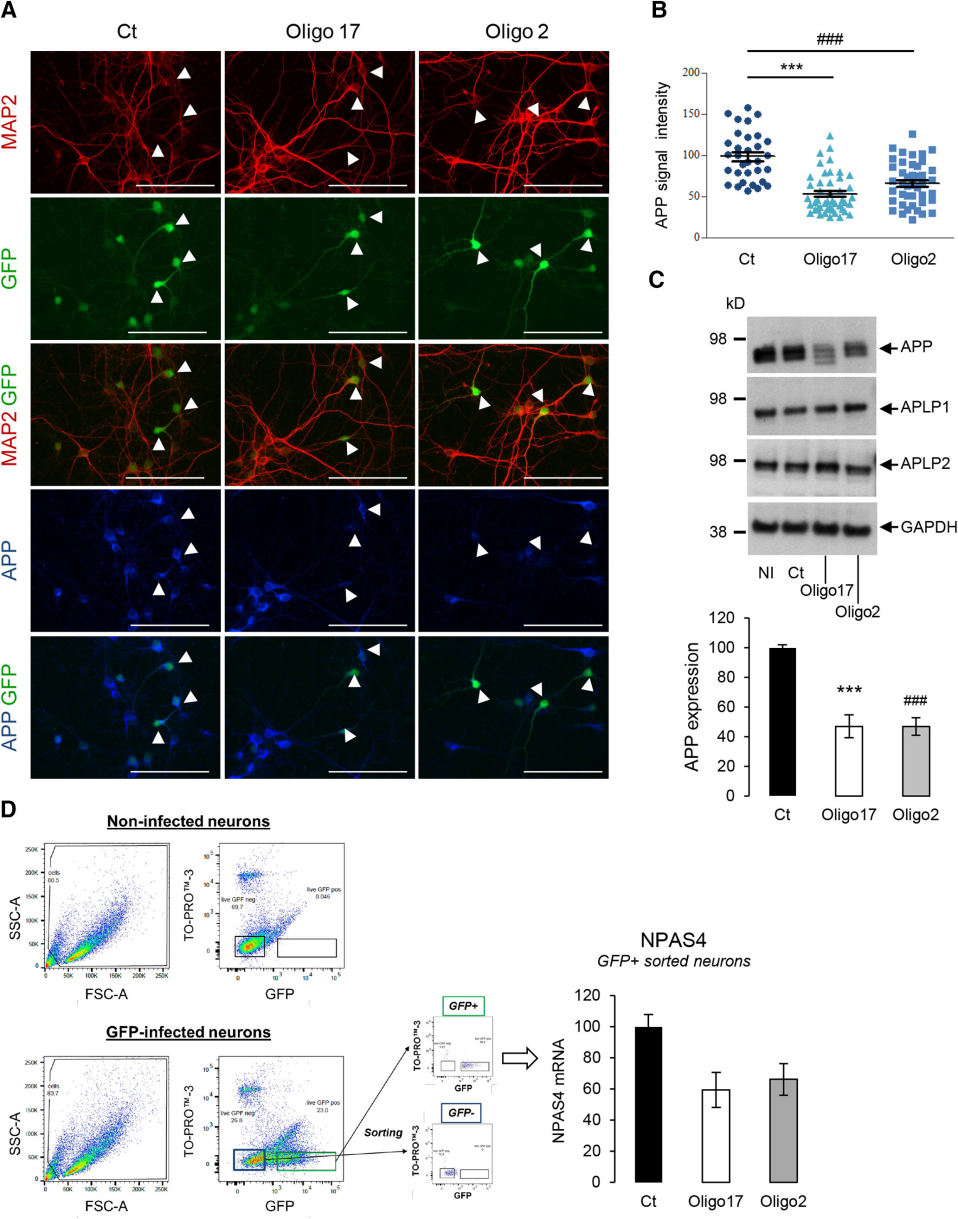
Decreased NPAS4 expression in APP-silenced primary neurons. APP was knock-down by CRISPR-Cas9 approach in primary neurons cultures. The infectivity and toxicity of lentiviral CRISPR-Cas9 vectors are detailed in Extended Data Figure 3-1. ***A***, APP expression characterization in neurons by immunostaining. Cortical neurons were infected at DIV1 with lentiviruses expressing sgRNAs (Oligo2, Oligo17) or not (Ct). All lentiviruses harbor a GFP expression cassette. Cultures were immunostained for MAP2 (red), APP (blue) and DAPI (light blue) at DIV7. Arrowheads indicate the position of GFP-positive (infected) neurons in each condition. Scale bar: 100 µm. ***B***, Quantification of APP signal in GFP-positive neurons by ImageJ. At least 33 neurons were quantified in two independent experiments for each condition (*n* = 33 *N* = 2). Results (mean ± SEM) are given as percentage of control (Ct); ###*p* < 0.001 (Ct vs Oligo 2) and ***p < 0.001 (Ct vs Oligo17); Kruskal–Wallis test and Dunn’s multiple comparison test. ***C***, upper panel, Representative Western blots showing APP, APLP1, APLP2, and GAPDH protein levels in cortical neurons at DIV7 infected in the same conditions as in Figure 1*A*. NI = non-infected. Lower panel, Quantification of APP expression in total cell lysates measured by Western blotting. Results (mean ± SEM) are given as percentage of control (Ct); ***p < 0.001 (Ct vs Oligo17), ###*p* < 0.001 (Ct vs Oligo 2), ANOVA and Bonferroni’s multiple comparison test (*n* = 6, *N* = 3). ***D***, Sorting of GFP-expressing neurons (FACS). Scatter plots (FSC vs SSC, left panels) of non-infected and GFP-expressing cells are shown. Dot plots (TOPRO-3, far red vs GFP, right panels) were used to gate (green rectangle) GFP-positive/TOPRO-3 negative cells. RNA was extracted from these cells and NPAS4 mRNA level was quantified by qPCR. Results were obtained from pooled samples (four wells of 4 cm^2^ each) for each condition (Ct, Oligo2 and Oligo17). Quantification was conducted on two independent experiments (*N* = 2). Results (mean ± SEM) are expressed as percentage of Ct.

The mild APP-dependent transcriptional regulations we observed are in line with the mild phenotype of APP knock-out mice (Müller et al., 1994; Zheng et al., 1995). Still, APP-dependent gene regulations that occur in the close-up could be hidden in the long-term by functional redundancies with other members of the APP family (Shariati and De Strooper, 2013). In agreement, APP−/− phenotype is better unraveled by acute downregulation of APP in the brain (Senechal et al., 2007). We performed a knock-down of APP expression in neurons with a lentiviral-based CRISPR-Cas9 genome editing approach (Jinek et al., 2012) to test the consequence of acute APP downregulation on NPAS4 expression. Nearly ∼50% of the cells were infected and no lentiviral toxicity was measured under our conditions (Extended Data Fig. 3-1). Only neuronal cells were infected, reflecting the tropism of the viral particles for neurons. APP expression was analyzed by ICF (Fig. 3*A*). Measurement of the intensity of APP signal in infected (GFP-positive) neurons (Fig. 3*B*) indicated a strong decrease in APP expression (40–50%) in neurons infected with CRISPR-Cas9 viruses targeting APP exon1 and exon 2 (Oligo2 and 17 sgRNA, respectively). This was confirmed by Western blotting (Fig. 3*C*). Importantly, expression of APLP1 and APLP2 was not altered in neurons infected under the same conditions, indicating that off target editing of APP did not occur in our experimental setup. To measure the expression of NPAS4 selectively in GFP-positive (knock-down) neurons, we sorted GFP-positive neurons by flow cytometry (Fig. 3*D*). TO-PRO-3 staining was used as a viability marker to exclude dead cells from the analysis. The sorting parameters were set by using non-infected neurons (GFP negative) and neurons expressing GFP (GFP positive) as standards. NPAS4 mRNA was readily decreased in neurons infected with Oligo2-expressing and Oligo17-expressing lentiviruses (GFP positive). Thus, acute APP knock-down resulted in decreased NPAS4 mRNA levels, confirming the APP-dependent NPAS4 transcriptional regulation observed in APP−/− neurons.

10.1523/ENEURO.0322-19.2020.f3-1 Extended Data Figure 3-1 Infectivity and toxicity of lentiviral CRISPR-Cas9 vectors. ***A***, Cortical neurons were infected at DIV1 with lentiviruses expressing sgRNAs (Oligo2, Oligo17, or CRISPR-*NPAS4*) or no sgRNA (Ct), SpCas9, and GFP. Cultures were immunostained for MAP2 (red) and DAPI (light blue) at DIV7. Scale bar = 400 µm. ***B***, Quantification of GFP+ neurons (GFP+/MAP2+) in total neuron population (MAP2+) after lentiviral CRISPR-Cas9 infection with control (Ct), Oligo2, Oligo17, or CRISPR-*NPAS4*. At least five fields were analyzed for each lentiviral vector in two independent experiments (*n* ≥ 5, *N* = 2). Results are expressed as percentage of GFP+/MAP2+ cells in total MAP2+ cells (mean ± SEM); n.s. = non-significant, Kruskal–Wallis test and Dunn’s multiple comparison test. ***C***, Measurement of LDH activity released after infection (DIV7) of primary neuron with control (Ct), Oligo2, Oligo17, or CRISPR-*NPAS4* at DIV7 lentiviral vectors. Background LDH release was determined in non-infected control cultures (NI). Results were expressed as percentage of total LDH release measured in non-infected control cultures (NI) in two independent experiments (*n* = 12, *N* = 2). Download Figure 3-1, TIF file.

### APP deficiency increases markers associated to GABAergic transmission

**Figure 4.**
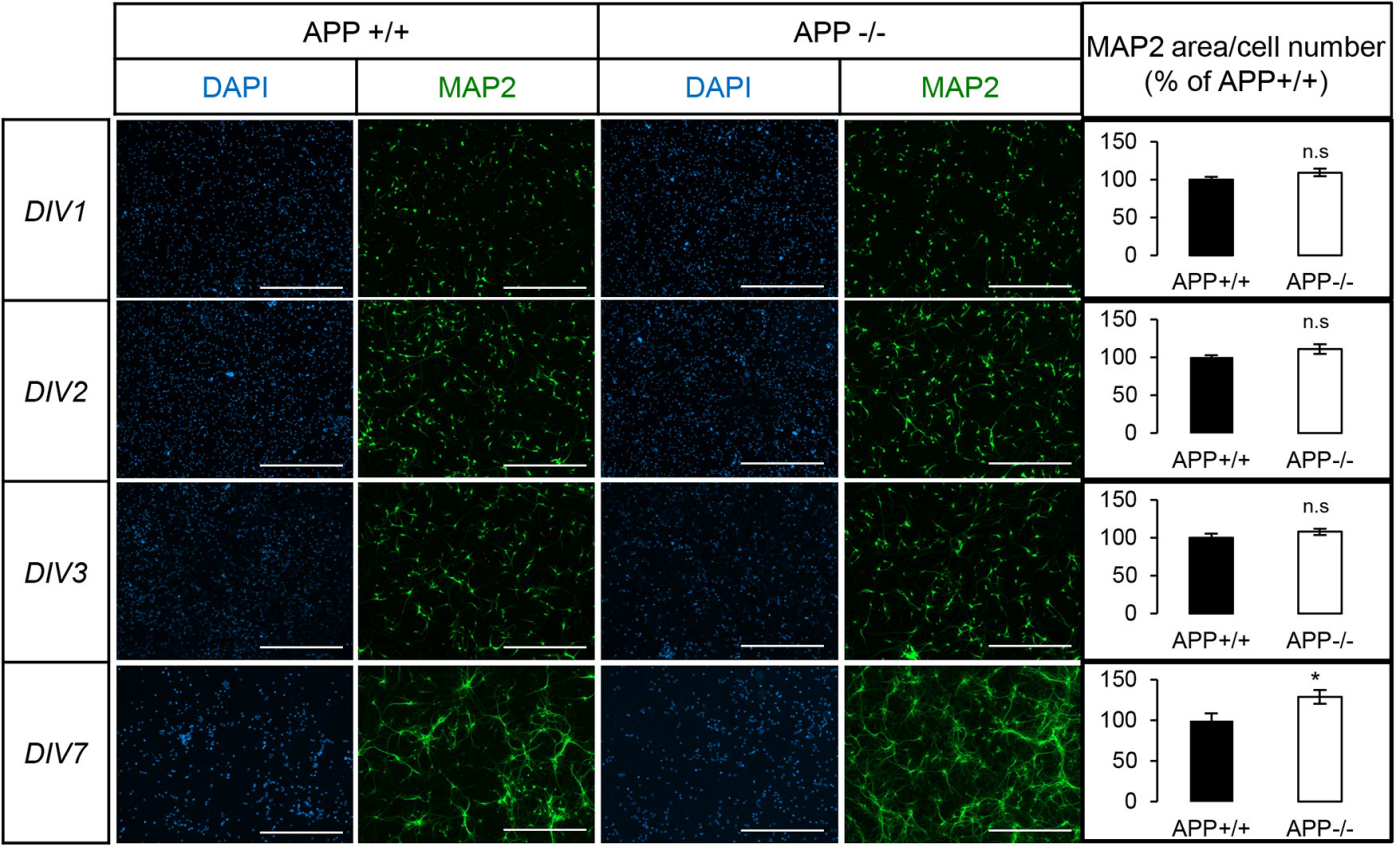
Altered neurites arborization of APP-deficient neurons during in vitro maturation. Cortical APP+/+ or APP−/− were stained against the neuron-specific marker MAP2 and the nuclear dye DAPI at different stages of maturation (DIV1–DIV3 and DIV7). Scale bar: 400 µm. Quantification by ImageJ of MAP2 signal area normalized to the number of neurons at DIV1–DIV3 and DIV7. Quantifications were from three fields of at least six coverslips from APP+/+ and APP−/− neurons, in three independent experiments (*N* = 3). Results (mean ± SEM) are expressed as percentage of control (APP+/+); *p = 0.0293, Mann–Whitney test. n.s. = non-significant.

**Figure 5.**
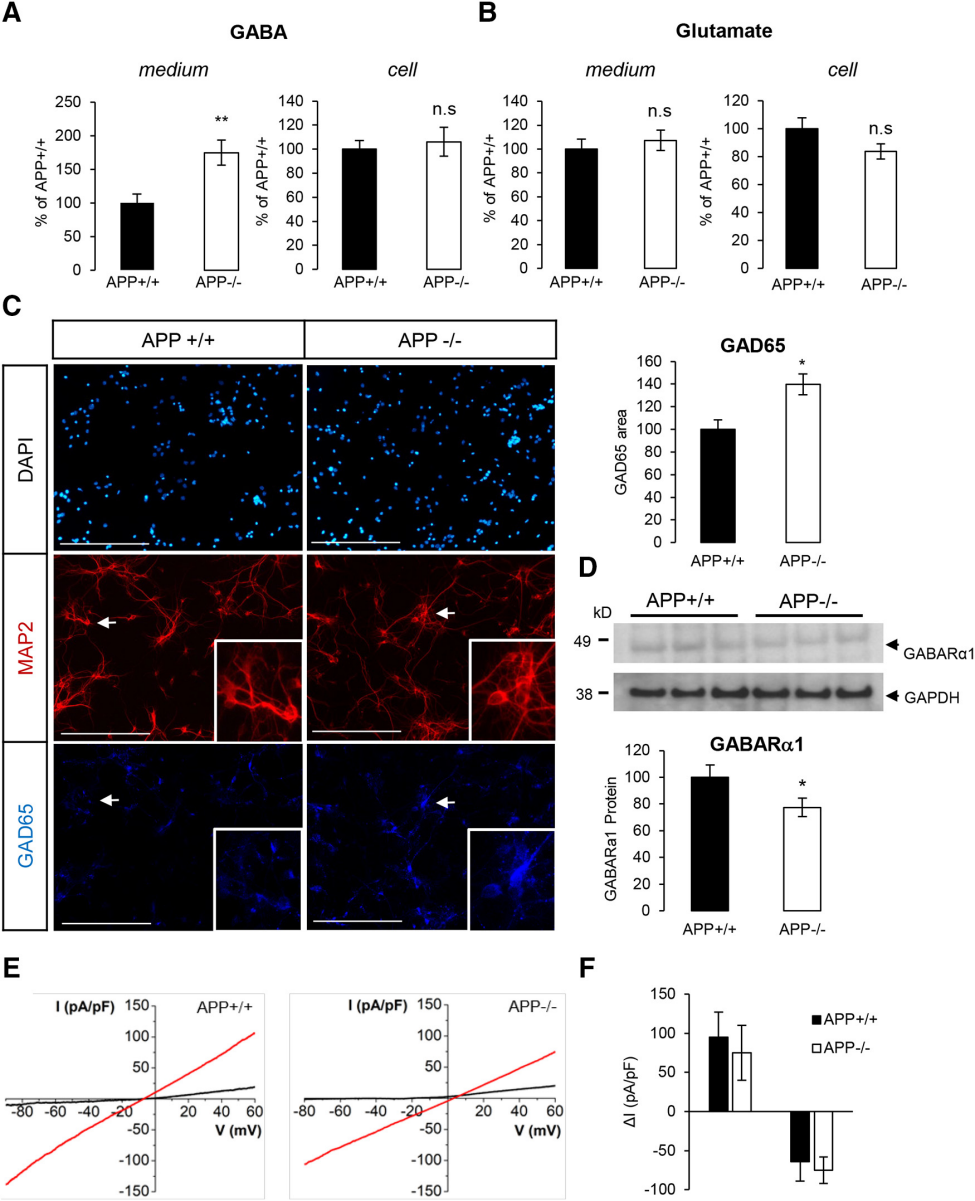
GABAergic markers and GABAergic transmission in APP knock-out neurons. ***A***, Quantification of GABA in culture medium and cell extracts of APP+/+ and APP−/− primary neurons at DIV7. Results (mean ± SEM) are expressed as percentage of APP+/+ (*n* = 20, *N* = 3); **p = 0.0024, n.s. = non-significant, Student’s *t* test. ***B***, Quantification of glutamate in culture medium and cell extracts of APP+/+ and APP−/− neurons at DIV7. Results (mean ± SEM) are expressed as percentage of APP+/+ (*n* = 16, *N* = 3); n.s. = non-significant, Student’s *t* test. Glutamate responses measured in APP−/− neurons are shown in Extended Data Figure 5-1. ***C***, Cortical APP+/+ and APP−/− neurons at DIV7 were immunostained for the neuron-specific marker MAP2 and GAD65. Arrows indicate MAP2/GAD65-positive neurons, shown at higher magnification (insets). The expression profile of the GAD65 GABAergic marker in APP+/+ and APP−/− neurons is detailed in Extended Data Figure 5-2. GAD65 signal (five fields per coverslip) was normalized for quantification to the number of cells in the area (histogram on the right). At least two coverslips were quantified for each group (APP+/+ and APP−/−) in two independent experiments (*N* = 2). Results (mean ± SEM) are given as percentage of control (APP+/+). Scale bar: 200 µm; *p = 0.0220, Mann–Whitney test. ***D***, Neurons harvested at DIV7 and cell extracts analyzed by Western blotting for GABARα1 and GADPH expression. Quantification of GABARα1 was normalized to GAPDH expression. Results (mean ± SEM) are expressed as percentage of Ct (*n* = 5, *N* = 2); *p = 0.0197, Student’s *t* test. ***E***, Representative I-V traces (from −90 to +60 mV repeated every 0.1 s) through APP+/+ (***A***) and APP−/− (***B***) neurons, in the presence 100 μm GABA (red traces). ***F***, Pooled data of whole-cell current (at +50 and −50 mV) evoked by 100 mm GABA, through APP WT and KO neurons. Each column represents mean ± SEM of *n* = 8 cells.

NPAS4 is an ITF induced by neuronal activity. The downregulation of NPAS4 expression observed in the absence of APP could reflect an impairment in the establishment of a functional network, leading to defects in basal neuronal activity. APP was reported to modulate neurite outgrowth and synapse formation (Priller et al., 2006; Young-Pearse et al., 2007; Tyan et al., 2012; Billnitzer et al., 2013). We analyzed neuronal arborization by measuring the area of the neuron-specific MAP2 signal per cell from DIV1 to DIV7 (Fig. 4). Neurite extension observed in APP+/+ and APP−/− neurons was not significantly different at DIV1 and DIV3. Strikingly, the absence of APP significantly increased MAP2 signal at DIV7, indicating the importance of APP for in neurite arborization and formation of a functional network. This observation reinforced the possible involvement of NPAS4 in APP physiological function. NPAS4 activity scales the neuronal network by controlling the balance of excitatory and inhibitory inputs on postsynaptic neurons (Lin et al., 2008; Bloodgood et al., 2013; Spiegel et al., 2014). We measured the amount of two neuromediators, GABA (released at inhibitory synapses) and glutamate (released at excitatory synapses) in the medium and in the cells of primary neurons at DIV7 (Fig. 5*A*,*B*). The concentration of GABA was increased by 83% in the medium of APP−/− neurons (Fig. 5*A*). No significant change in glutamate concentration (cell or medium) was observed in APP−/− neuronal cultures when compared with APP+/+ (Fig. 5*B*). In line with this observation, we measured only very subtle changes in glutamate responses in APP−/− neurons when compared with APP+/+ (Extended Data Fig. 5-1), pointing toward a specific impairment in GABA-dependent signaling in the absence of APP. GABA is synthetized in inhibitory neurons by the GAD enzymes (GAD_65_ and GAD_67_). GAD_65_ is active at nerve terminals and synapses. We observed that GAD_65_ signal is increased in APP−/− neurons when compared with APP+/+ (Fig. 5*C*). This is not caused by an increase in the relative number of GAD_65_-positive neurons in APP−/− cultures (Extended Data Fig. 5-2), but likely to an increase in GAD65 expression in GABAergic neurons. To further address the effect of APP deficiency on GABAergic synaptic markers, we first quantified the expression of GABARα1, a GABA_A_ receptor subunit predominantly expressed during neuronal development. We found a slight but significant decrease in GABARα1 in APP−/− neurons (Fig. 5*D*). To evaluate whether the activity of GABA receptors was defective in physiological conditions, cortical neurons at DIV7 were voltage-clamped at –60 mV. To prevent neuronal activity, experiments were performed in the presence of 1 μm TTX, 10 μm CNQX, 20 μm D-AP5, and 100 nm CGP55845 to inhibit Na^+^ voltage-dependent channels, AMPA, NMDA, and GABA_B_ receptors, respectively. Current-voltage (IV) curves were generated by evoking the current with a voltage ramp stimulus from –90 to +60 mV, and the response to 100 μm was studied (Fig. 5*E*). Whole-cell currents evoked at –50 or +50 mV by 100 μm GABA were similar in APP+/+ as in APP−/− neurons (Fig. 5*F*). This patch-clamp investigation did thus not reveal any functional decrease in GABA_A_ receptors, suggesting that the minor decrease in expression of GABARα1 subunit was compensated.

10.1523/ENEURO.0322-19.2020.f5-1 Extended Data Figure 5-1 Glutamate responses in APP−/− neurons measured by calcium imaging. Neuronal activity was measured at DIV7 by calcium imaging. ***A***, left panel, Different calcium responses were observed after stimulation with 50 µm glutamate and classified as described by Pickering et al. (2008) between neuronal and non-neuronal responses. To note *x*-axis graduation corresponds to 20 s. Right panel, The proportion of cells displaying type 1, 2, or 3 response was quantified in three independent experiments (*n* = 9, *N* = 3); n.s. = non-significant. Student’s t *test*. ***B***, Normalized fluorescence trace (mean ± SEM) measured in APP+/+ and APP−/− neurons upon perfusion for 150 s with 50 µm glutamate. The AUC was quantified for 50 neurons per coverslips. A total of nine coverslips for each genotype was recorded in three independent experiments (*N* = 3). The graph on the right shows AUC expressed as percentage of control (APP+/+); *p = 0.0106, Student’s *t* test. Download Figure 5-1, TIF file.

10.1523/ENEURO.0322-19.2020.f5-2 Extended Data Figure 5-2 GAD65-positive neurons in primary cortical cultures. ***A***, Primary culture of cortical neurons after at DIV7. Cultures were immunostained with the neuron-specific protein MAP2 (red), GAD65 (dark blue), and DAPI (light blue). Representative 20× micrographs show GAD65-positive neurons (white arrowhead) and GAD65-negative neuron (green arrowhead). ***B***, Images (20× objective) were quantified (10 fields per coverslip for each genotype) in three independent cultures (*n* = 30, *N* = 3). Results (mean ± SEM) are expressed as percentage of GAD65+ MAP2+ cells (GAD65+ neurons) among all MAP2+ cells (neurons); n.s. = non-significant, Mann–Whitney test. Scale bar = 20 µm. Download Figure 5-2, TIF file.

**Figure 6.**
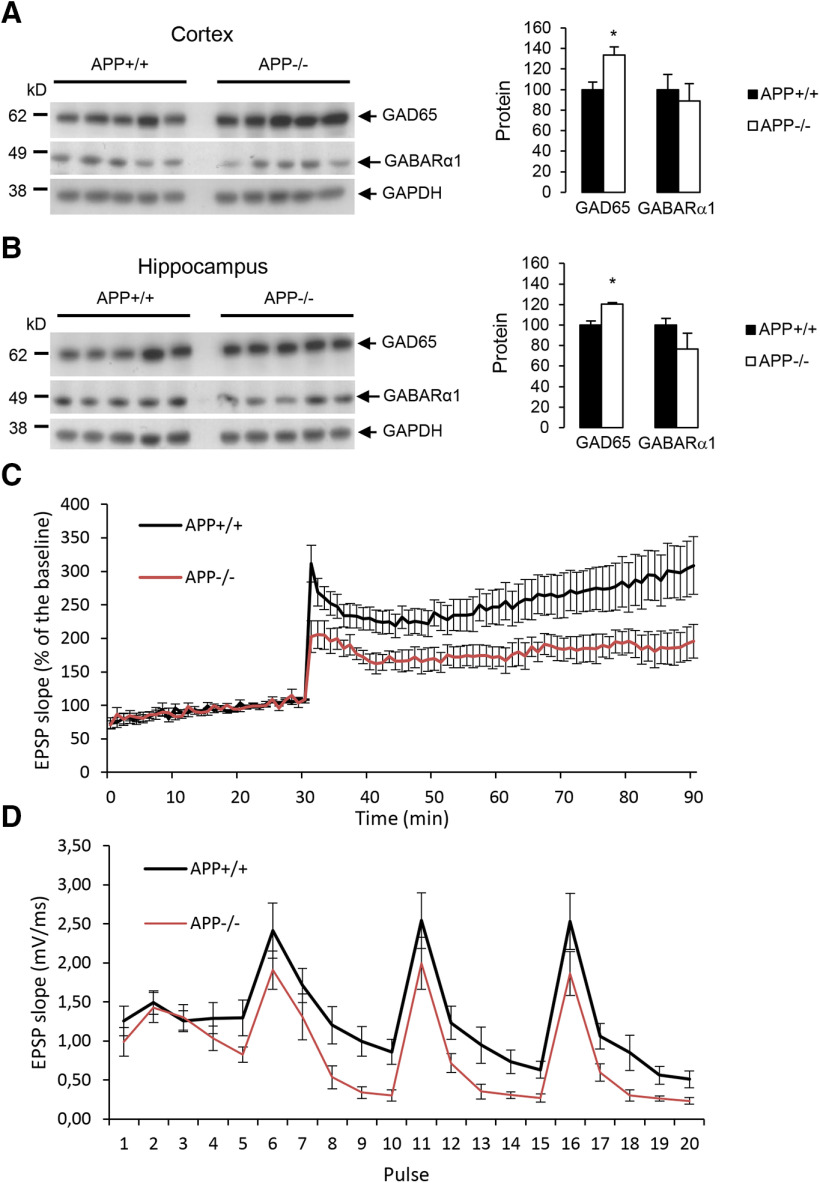
GABAergic markers and LTP in adult mice. ***A***, left panel, Western blot analysis of GAD65, GABARα1, and GAPDH expression in cortex of three-month-old APP+/+ and APP−/− mice (*N* = 5). Right panel, Quantification of GAD65 and GABARα1 were normalized to GAPDH expression. Results (mean ± SEM) are expressed as percentage of APP+/+ (*N* = 5); *p = 0.0166, Student’s *t* test. ***B***, left panel, Western blot analysis of GAD65, GABARα1, and GAPDH expression in hippocampus of three-month-old APP+/+ and APP−/− mice (*N* = 5). Right panel, Quantification of GAD65 and GABARα1 were normalized to GAPDH expression. Results (mean ± SEM) are expressed as percentage of APP+/+ (*N* = 5); *p = 0.0404, Student’s *t* test. LTP was measured in hippocampal SC-CA1 pathway from APP+/+ (*n* = 9) and APP−/− mice (*n* = 8). The input-output relationship between fEPSP measured in CA1 stratum radiatum and the intensity of SC stimulation is depicted in Extended Data Figure 6-1. ***C***, fEPSP slopes measured during TBS (*p* < 0.05; two-way repeated measurements ANOVA, Bonferroni). ***D***, fEPSP slopes measured before and after TBS. Results expressed in proportion of the baseline response (100%; *p* < 0.05; two-way repeated measurements ANOVA, Bonferroni).

Finally, we evaluated the impairment in GABAergic markers in the brain of APP−/− mice. We quantified the expression of GAD65 and GABARα1 in cortices and hippocampi of three-month-old mice. A significant increase in GAD65 expression was observed both in cortex (Fig. 6*A*) and in hippocampus (Fig. 6*B*) of APP−/− mice, and decreased GABARα1 levels were measured especially in the hippocampus. We analyzed CA3-to-CA1 synapse plasticity by extracellular recordings on hippocampal slices from adult APP+/+ and APP−/− mice. SCs were stimulated and fEPSP were recorded in the stratum radiatum of CA1 region. We observed that the relationship between the stimulus intensity and the fEPSP slope was similar in slices from both genotypes (Extended Data Fig. 6-1*A*). To investigate LTP, we stimulated the SC pathway with a TBS consisting of four bursts of five pulses (given at 100 Hz) separated by 200 ms. In slices from APP+/+ animals, TBS induced a large increase of the fEPSP response size that decayed over the first 20 min to a plateau level persisting up to the end of the experiment. In APP−/− mice, LTP was significantly reduced (Fig. 6*C*). Typically, 60 min after the TBS, LTP was reduced by half. The response to TBS itself was actually modified: the second, third and fourth bursts of stimulation were globally decreased in APP−/− compared with APP+/+, and within each of the four bursts of 5 pulses, the responses to the third, fourth and fifth stimuli decreased more in slices form APP−/− mice than in APP+/+ (Fig. 6*D*).

10.1523/ENEURO.0322-19.2020.f6-1 Extended Data Figure 6-1 LTP in hippocampal SC-CA1 pathway in APP−/− mice. Excitatory postsynaptic potentials measured in hippocampal CA1 region of brain slices from APP+/+ (*N* = 9) and APP−/− mice (*N* = 8). ***A***, The input-output relationship between fEPSP measured in CA1 stratum radiatum and the intensity of SC stimulation is represented. No significant difference between APP+/+ and APP−/− was observed. Download Figure 6-1, TIF file.

### Silencing NPAS4 mimics APP deficiency in neurons

**Figure 7.**
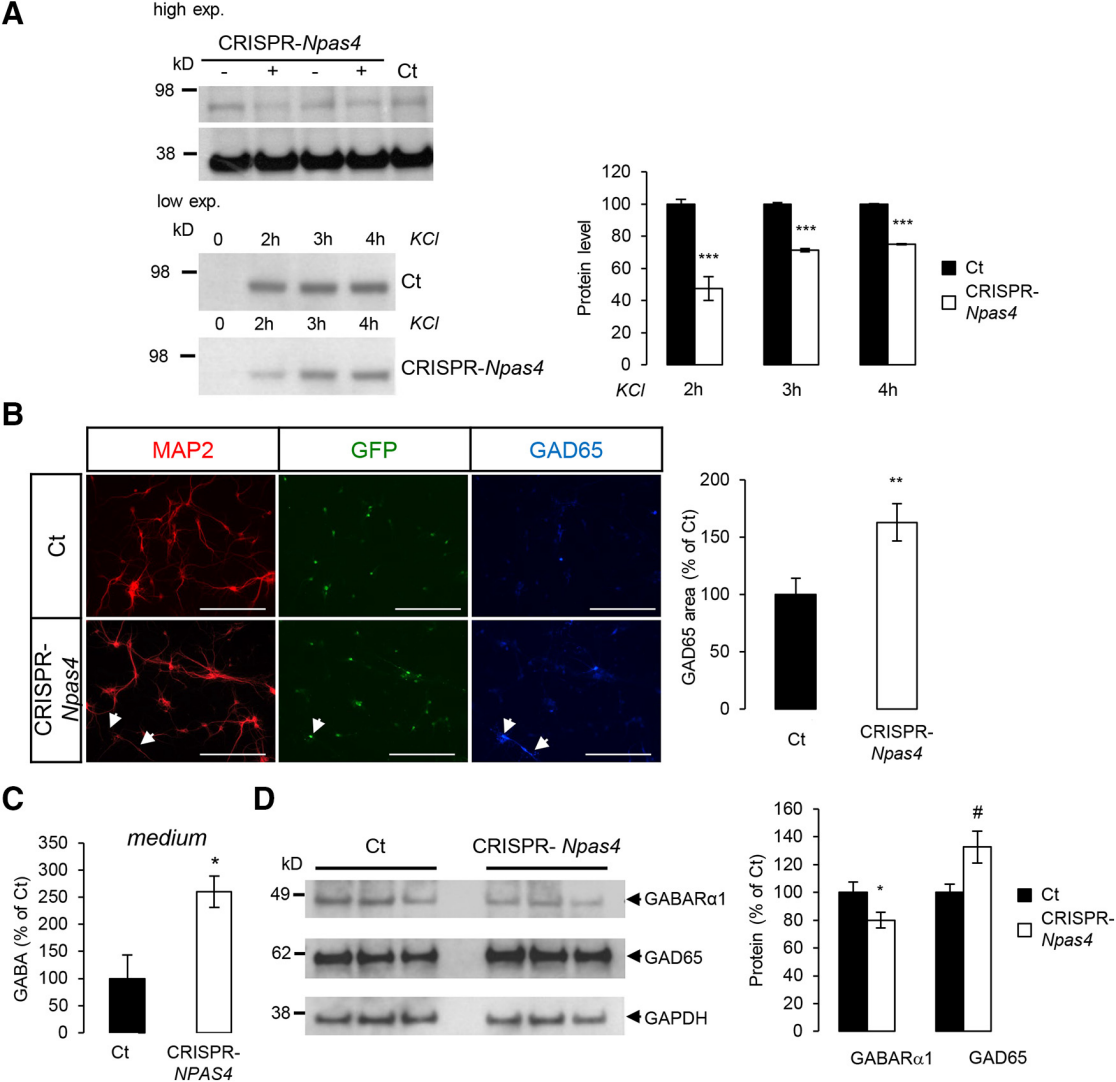
NPAS4 silencing by CRISPR-Cas9 mimics cell phenotype observed in APP-deficient neurons. Changes on inhibitory (GABA) synapses was analyzed after *NPAS4* silencing ***A***, left panel, Cortical neurons infected with CRISPR-Cas9 lentivirus targeting *NPAS4* gene (CRISPR-*NPAS4*) show reduced NPAS4 levels as measured by Western blotting (high exposure). Same experiments were conducted after membrane depolarization with 50 mm KCl. NPAS4 accumulations was detectable by Western blotting at low exposure. Viruses without sgRNA were used as controls (Ct). Right panel, Quantification of NPAS4 protein level after 2, 3, and 4 h of KCl depolarization. Results (mean ± SEM) are expressed as percentage of non-treated controls Ct (*N* = 2); ***p < 0.0001 Student’s *t* test. ***B***, Cortical neurons infected with CRISPR-*NPAS4* lentiviruses at DIV1 were immunostained against MAP2 and GAD65 at DIV7. Quantification of GAD65 signal was normalized to the number of cells (five fields per coverslip, two coverslips for each genotype in two independent experiments (*N* = 2). Results (mean ± SEM) are given as percentage of control (Ct). Scale bar: 200 µm; **p = 0.0024. Mann–Whitney test. ***C***, Quantification of GABA in culture medium at DIV7 of infected control neurons (Ct) and CRISPR-*NPAS4*-infected neurons. Results (mean ± SEM) are expressed as percentage of Ct (*n* = 5, *N* = 2); *p = 0.0146, Student’s *t* test. ***D***, Neurons harvested at DIV7 and cell extracts analyzed by Western blotting for GABARα1, GAD65, and GADPH expression. Quantification of GABARα1 and GAD65 were normalized to GAPDH expression. Results (mean ± SEM) are expressed as percentage of Ct (*n* = 8, *N* = 3); *p = 0.049, #*p* = 0.0247, Student’s *t* test.

We used the CRISPR-Cas9 approach to directly silence *NPAS4* expression in neurons and analyze whether NPAS4 deficiency could recapitulate a major trait observed in APP−/− neurons, i.e., the upregulation of GABA release and modification of GABAergic markers. The efficiency of CRISPR-Cas9 editing is hard to evaluate by quantifying the mRNA levels of the target gene, since decrease in mRNA could only occur if nucleotide insertions by non-homologous end-joining results in nonsense-mediated RNA decay. Commercially available antibodies poorly detect NPAS4 in basal conditions, but we could still observe that NPAS4 protein was diminished on silencing (CRISPR-*NPAS4* condition; Fig. 7*A*). We further decided to check the downregulation of *NPAS4* gene expression by measuring NPAS4 protein on depolarization by KCl (Lin et al., 2008). In that condition, we found that CRISPR-Cas9-induced silencing resulted in a decrease in NPAS4 by ∼50% (Fig. 7*A*). This is comparable to the decrease in NPAS4 mRNAs measured in APP−/− neurons at DIV7 (Fig. 1*D*). As for CRISPR-Cas9 lentiviral vectors targeting APP, we did not observe toxic effects related to viral transduction of primary neurons (Extended Data Fig. 3-1*C*). Strikingly, like APP-deficient primary neurons (Fig. 5*C*) or brains of APP−/− mice (Fig. 6), NPAS4-deficient neurons showed an increase in GAD65 staining (Fig. 7*B*,*C*) and GAD65 protein expression (Fig. 7*D*). Accordingly, a 2.5-fold increase in GABA concentration was measured in the medium of primary neurons infected with CRISPR-Cas9 *NPAS4* lentiviruses (Fig. 7*C*). The expression of GABA receptor subunit α1 (GABARa1) was decreased after *NPAS4* knock-down (Fig. 7*D*), to the same extent as the decrease observed in in APP−/− primary neurons (Fig. 5*D*).

## Discussion

One major function of APP is to control synaptic formation, transmission and plasticity (Müller et al., 2017). We showed here that APP deficiency in cortical neurons impairs the balance between excitatory and inhibitory synaptic markers, and increases GABAergic transmission. This process relies on the activity-dependent transcription factor NPAS4. We initially identified the NPAS4 IEG as downstream APP target by a non-biased transcriptome analysis. The APP-dependent transcription of NPAS4 involves its extracellular domain (sAPPα) and is activated by AICD. APP appears thus to exert a fine tuning of inhibitory synapses in neuronal network. Its absence enhances, through the downregulation of NPAS4, the production and the release of GABA.

### APP-dependent expression of NPAS4 in differentiated neurons

The transcriptome analysis of APP+/+ versus APP−/− neurons at embryonic day 18 (E18–DIV0) and at different stages of primary cortical neuron differentiation (DIV3–DIV7) indicated that the transcriptional changes in the absence of APP were moderate. This unexpected result is in line with a comparative transcriptome study of APP family members in the adult mouse cortex (Aydin et al., 2011). Subtle effects of APP deficiency on the transcriptome could be due to functional redundancies with other APLPs that display similar functions and signaling properties (Shariati and De Strooper, 2013). We did not measure any changes in APLP1 and APLP2 expression in our APP−/− models, in agreement with studies done on total brain extracts (Zheng et al., 1995) or in primary cortical neurons (White et al., 1998). Transcriptional modifications we measured are thus related to the absence of the APP protein per se APP-dependent transcriptional regulations likely act by fine-tuning the expression of classes of gene involved in neuronal pathways rather than robustly regulating single target genes. We found that the expression of the NPAS4 ITF is downregulated in the absence of APP. This particularly at DIV7, when primary neurons start to form a functional network. NPAS4 downregulation was observed it in APP−/− primary neurons and on acute APP knock-down by a CRISPR-Cas9 approach, establishing a link between APP and NPAS4 transcription. Regarding the possible molecular mechanisms involved here in APP-dependent gene transcription, we found that NPAS4 expression is activated in neurons by AICD expression. It correlates with the fact that DIV7 corresponds to the differentiation stage where AICD is peaking in primary cortical neurons. (Kimberly et al., 2005). However, previous studies showed the secreted ectodomain (sAPPα) is sufficient to rescue functional defects in APP KO mice (Ring et al., 2007; Weyer et al., 2014). We found that treatment of APP+/+ neurons (and not APP−/− neurons) with sAPPα significantly increased NPAS4 mRNAs. This observation indicates that (1) the effects of sAPPα require the presence of endogenous APP, and (2) homophilic ectodomain interactions are likely to be involved. sAPP was suggested to promote its physiological effects by interaction with APP holoprotein (Milosch et al., 2014; Deyts et al., 2016). It is tempting to suggest here that interaction of sAPP with APP holoprotein present at the cell surface could induce the release of transcriptionally active AICD, but this hypothesis awaits further experimental evidence.

### Alteration of GABA release and GABA markers in APP-deficient neurons

In the absence of APP, we observed an increase in neuronal outgrowth and GAD65 signal, as well as increased GABA release in the medium of primary neurons. The increase in GAD65 signal was related to an increased signal in GAD65-positive neurons, not to an increased number of GAD65 neurons in our primary cultures. It would be useful to further investigate as to whether an increase in the numbers and functionality of GABAergic neurons occurs in the brain of APP−/− mice. To note, APP was reported to control neurogenesis in adult mice brain (Wang et al., 2014), as process that could account for the modification of neuronal subtypes observed in the absence of APP.

The increase-in GAD65 we observed was in line with increased levels of GABA in the culture medium. *In vivo* studies evidenced increased GABA levels in the brain of APP−/− mice (Lee et al., 2010). However, this elevation of GABA markers was concomitant to a downregulation in the GABARα1 receptor subunit. Recent study also reported that GABARα1 is particularly decreased in hippocampus of APP−/− mice (Chen et al., 2017), in line with our *in vivo* experiments. Still, patch-clamp experiments did not reveal any decrease in GABA_A_ receptors functionality, indicating that compensation may occur to circumvent the decrease in a GABA_A_ receptor subunit, and that GABAergic transmission per se is not significantly altered.

On the other hand, our results show that LTP is reduced in brain slices from APP−/− mice. This is in agreement with previous studies showing that the LTP induced by one TBS is reduced in APP−/− at the SC-CA1 synapses (Dawson et al., 1999; Seabrook et al., 1999; Ring et al., 2007) but not at the perforant path-granule cell (DG) synapse that we did not study here (Jedlicka et al., 2012), Such inhibition was however not observed by other investigators (Wang et al., 2017), possibly because they used a stronger stimulation protocol (three TBS). Interestingly, the analysis of the responses to the TBS itself is consistent with increased GABAergic release in APP−/− mice.

How could APP modulate GABA release? sAPPα is known to enhance LTP and it is sufficient to rescue the decrease of LTP observed in APP−/− mice (Ring et al., 2007; Taylor et al., 2008). Moreover, very recent studies showed that the sAPPα directly binds the GABA_B_ receptor subunit 1a (GABA_B_R1a), suppressing synaptic transmission and triggering short-term facilitation in hippocampal neurons (Rice et al., 2019). Such an effect of sAPPα could explain the smaller response observed in brain slices from APP−/− mice compared with APP+/+. Indeed, in APP−/− slices, a larger release of GABA would occur in the absence of GABA_B_R1a stimulation by sAPPα, therefore reducing the disinhibitory process observed between the first and the second burst of stimuli of the TBS (Larson and Munkácsy, 2015). This supports that APP synaptic function and APP-dependent synaptic transmission are mediated by the soluble sAPPα fragment. We suggest that the NPAS4 IEG is sensing the APP-dependent modulations of synaptic transmission. Our results also indicate that NPAS4 knock-down mimics APP deficiency on GAD65 levels and GABA measurements. The finding that APP functions in neuronal network might be mediated by NPAS4 is relevant to several evidences about the reported role of NPAS4 in neuronal network activity. NPAS4 possesses unique features among the IEGs (Sun and Lin, 2016): (1) it is only expressed in neurons; (2) it is activated selectively by neuronal activity; and (3) it has been shown to shape glutamatergic and GABAergic synaptic inputs. NPAS4 is implicated in a transcriptional program that regulates neuronal firing responses to excitatory transmission by enhancing inhibition (Lin et al., 2008), and is critical to keep neuronal firing in response to stimuli within normal levels (Spiegel et al., 2014). This exciting new field of investigation connecting APP function to ITFs sensing neuronal activity awaits further investigation.

### Possible relevance to the AD pathophysiology

APP plays a central role in the onset and progression of the amyloid pathology found in AD. Apart from producing Aβ, the precise contribution of the APP protein to the pathology is poorly understood. The impairment of APP function, either caused by familial AD (FAD) mutations or on neuronal aging, may contribute to neuronal dysfunctions occurring in the disease. In the mammalian brain, APP modulates dendritic complexity, synaptic functions, and synaptic plasticity (Müller et al., 2017). A reduction in dendritic length and branching as well as in total spine density was reported in old APP-deficient mice (Lee et al., 2010; Tyan et al., 2012). Aging is an important parameter related to APP functions in the brain. The phenotype related to APP deletion in the CNS is age dependent (Priller et al., 2006). Upon aging, impairments in learning and memory associated with deficits in LTP are observed in APP-deficient mice as shown here and in previous studies (Ring et al., 2007). Interestingly, the decline in memory performance and reduction in LTP observed in old mice and APP transgenic mice are mediated by the ionotropic GABA_A_ receptor (Yoshiike et al., 2008). This imbalance in neuronal excitatory/inhibitory transmission was also observed in the temporal cortex of AD patients, where significantly lower levels of GABA and glutamate were measured (Gueli and Taibi, 2013). These observations unambiguously indicate that changes in neurotransmission occur in AD (and even in aging brain) and point toward alteration of the inhibitory GABAergic transmission. Important points must be kept in mind here. First, GABAergic transmission shifts from excitatory to inhibitory during development (Ben-Ari, 2002), so the consequence of altered GABAergic transmission can be fully understood only in adult brain. Then, the molecular mechanisms underlying changes in inhibitory transmission in AD are complex. The GABAergic molecular actors are differentially affected by aging (Vela et al., 2003) or in AD mice models (Yoshiike et al., 2008). Decrease in GABARα1 has been reported in aging rodent brain and in the hippocampus of aged brains with AD (Mizukami et al., 1998), but this could be functionally compensated as shown herein. Our findings should further be addressed in AD mice models (expressing APP mutations) to complete the results obtained here in a loss-of-function model (APPKO). However, the hypothesis of an overall impairment of GABAergic transmission in AD is also supported by the increased risk for unprovoked seizures observed in individuals with AD compared with non-demented individuals of the same age (Friedman et al., 2012).

Finally, NPAS4 expression was found to decrease along with AD progression, particularly at Braak NFT stages (I–II) corresponding to lesions developed in transentorhinal/entorhinal cortex (Miyashita et al., 2014). We believe that our main observation, namely that APP deficiency in neurons is integrated by the activity-dependent NPAS4 IEG and affects the balance of inhibitory and excitatory neuronal inputs, provides new insight to understand the role of APP in synaptic activity, but also a mechanistic frame to further explore the impairments of network activity in AD.

## Synthesis

Reviewing Editor: Menno Witter, Norwegian University of Science and Technology Decisions are customarily a result of the Reviewing Editor and the peer reviewers coming together and discussing their recommendations until a consensus is reached. When revisions are invited, a fact-based synthesis statement explaining their decision and outlining what is needed to prepare a revision will be listed below. The following reviewer(s) agreed to reveal their identity: Karunya Kandimalla.

The manuscript shows that downregulation of APP reduces expression of NPAS4 and increases the expression of GAD65. Although these are interesting findings, the results are mainly from immature in vitro neuronal cultures, very descriptive and without proper validation or assessment of the functional consequences of these changes in vitro and in vivo.

The title “Amyloid Precursor Protein (APP) controls excitatory/inhibitory synaptic inputs by regulating the transcriptional activator Neuronal PAS Domain Protein 4

(NPAS4)” is an overinterpretation of the results, since “excitatory/inhibitory synaptic inputs” are not measured. In addition, the causal role of NPAS4 is not explored since mechanistic manipulations restoring the levels of NPAS4 levels are not implemented. Overall, the results are preliminary and do not support the notion that NPAS4 regulates APPKO effects on synaptic function.

We therefore feel that additional (neurophysiological) characterizations are required to support the claims made in the paper.

Additional comments:

1) When you submit a revised version you need to submit a graphical abstract

2) Define NPAS4 as an inducible transcription factor in the abstract.

3) Line 306, define better “APLP1 or APLP2 levels were observed”. Were the levels changed as concluded? Please clarify and describe properly.

4) It seems Figures 1A and 1C contain no significant changes for adjusted p values. “The Benjamini-Hochberg multiple correction test did not reveal any robust differential gene expression (adjusted p-value <0.05) excepted for APP (positive control).” But, fold changes are provided. Please add p values to the graph to alert that these fold changes are not significant.

5) Figure 1B, please add p values for KEGG pathways.

6) Line 364, Figure 2D, “AICD expression in APP-/- neurons did not modify NPAS4 mRNA levels (Fig. 2D)”. This figure clearly shows what it seems an upregulation of NPAS4 mRNA levels. This conclusion requires additional experiments. Overall this figure needs additional experiments, since Figures 2D and 2F shows similar inductions of NPAS4 mRNA independent of p values.

7) Figure 6 shows data from cortex and hippocampus of APP-/- mice, is NPAS4 reduced in APP-/- mice? What about the other findings in vitro? Excitatory/inhibitory synaptic inputs should be assessed in brain slices from APP-/- mice.

8) Line 295, please describe better the n numbers. “The number of samples (n) and the number of independent experiment per condition (N, biological replicates) are indicated in figure legends.” Is “n” the total number of samples, the number of samples per condition, or the number of samples per condition across the experiments? Please define independent experiment. It is unclear the term “independent experiment per condition” since an independent experiment should have all conditions.

9) Consider to condense the data into fewer tables

10) Extended data do not directly support their figures.

11) Edgar et al., 2002 reference was provided in the gene expression methods section. However, it was not found in the References section.

12) It appears that the microarray data used in this manuscript has been deposited in GEO. The exact source of the data is poorly described. The details were not clearly provided in the Methods section. It was not clear, if the authors were using the data that has been previously submitted to GEO or if the data was generated as part of the current study. In either case, the GEO series ID of the study should be provided in the main manuscript.

13) Consider to represent log2 fold changes while describing the differential expression results. It seems like the authors used linear fold changes instead.

14) The Figure legend consist of mistakes “Linear fold changes have been set at 1,25 , 1,5 etc.” The commas should be replaced with a decimal point.

15) KEGG pathway analysis was conducted to identify molecular pathways and the genes that are up and down regulated have been listed for the five most modified pathways. However, the authors should at least speculate on how they are connected to APP expression.

16) The authors should clearly describe the stereological techniques that were followed during quantification of immunostained neurons.

17) In addition to correlating NPAS4 expression with APP expression, the authors should follow the effects of APP deficiency on synaptic transmission using neurophysiological investigations. Otherwise, the manuscript conclusions are not fully supported.

18) There are several spelling mistakes in the manuscript, a thorough proof reading is required before it is acceptable for publication.
