# Supplementary figures and images for: Amyloid Precursor Protein (APP) Controls the Expression of the Transcriptional Activator Neuronal PAS Domain Protein 4 (NPAS4) and Synaptic GABA Release
Source: eNeuro. 2020 May 28;7(3):ENEURO.0322-19.2020. doi: 10.1523/ENEURO.0322-19.2020 (PMC7262005; doi:10.1523/ENEURO.0322-19.2020)

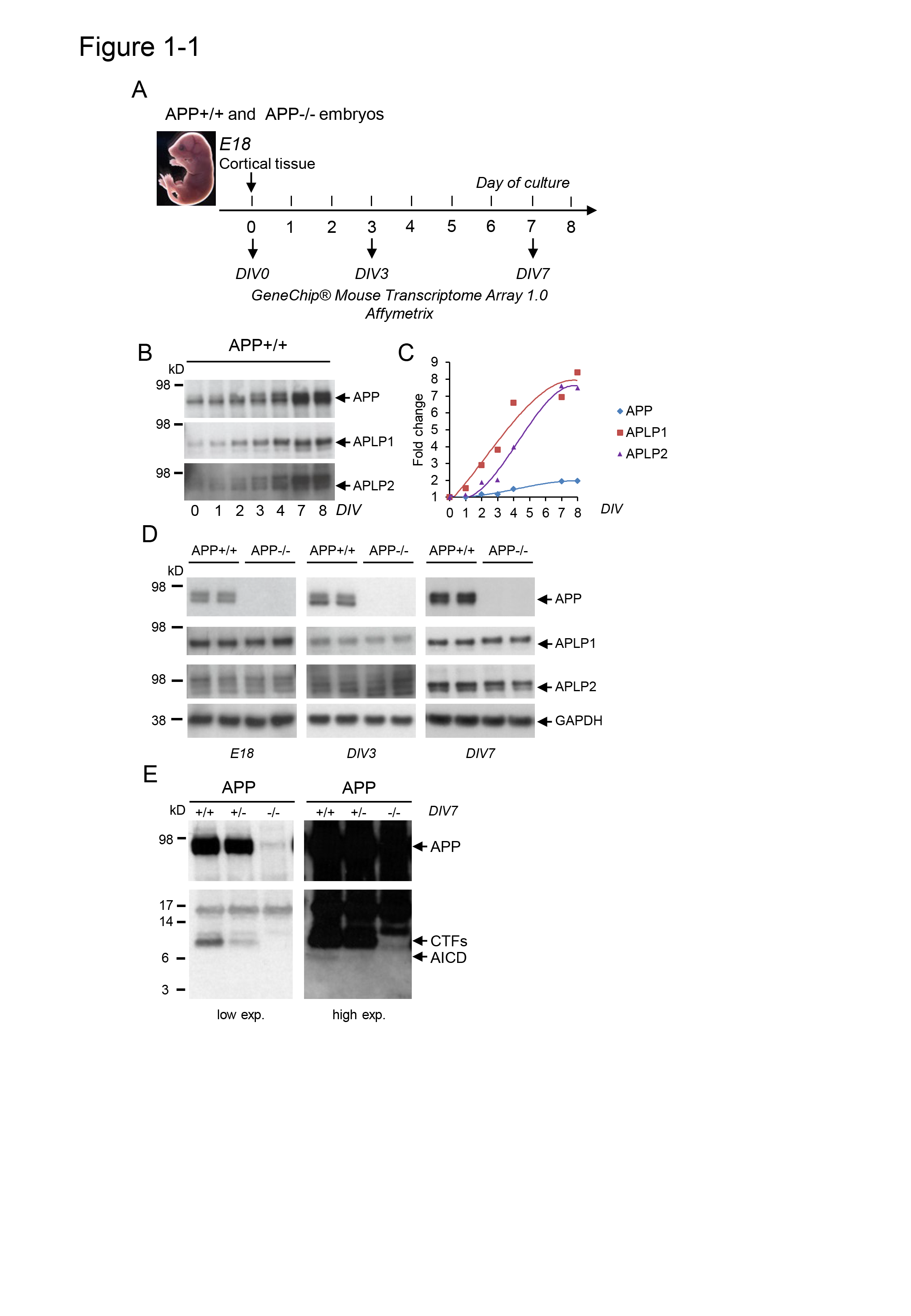

Supplement: Extended Data Figure 1-1 — Experimental workflow and model characterization. A, Experimental design used for the study (E18); neurons were cultured and experiments were mainly carried out after 3 and 7 DIV (DIV3 and DIV7). Transcriptome analysis was performed on embryonic cortex (E18) and at DIV3 or DIV7. B, APP, APLP1, and APLP2 expressions were analyzed by Western blotting at the indicated days of culture in APP+/+ neurons. C, Quantification of APP, APLP1, and APLP2 protein expression over time in APP+/+ neurons. Accumulation is represented as fold change over the signal measured at day 0. Quantification was performed from one neuronal culture. D, APLP1 and APLP2 expressions are not modified in cortical tissue at E18 and primary neuron cultures at DIV3 and DIV7 in absence of APP. Expression of APP, APLP1, APLP2 was analyzed by Western blotting of cells lysates from APP+/+ and APP−/− primary neuron cultures. E, Samples from primary cultures at DIV7 (APP+/+, APP+/−, and APP−/− neurons) were probed (Western blotting) with an antibody directed against APP C terminus for APP C-terminal fragments (CTFs) and AICD. Low and high exposures of a typical blot are shown. Arrows indicate the expected position of APP holoprotein, APP CTFs, and AICD. Download Figure 1-1, TIF file. [file enu-eN-NWR-0322-19-s02.tif]

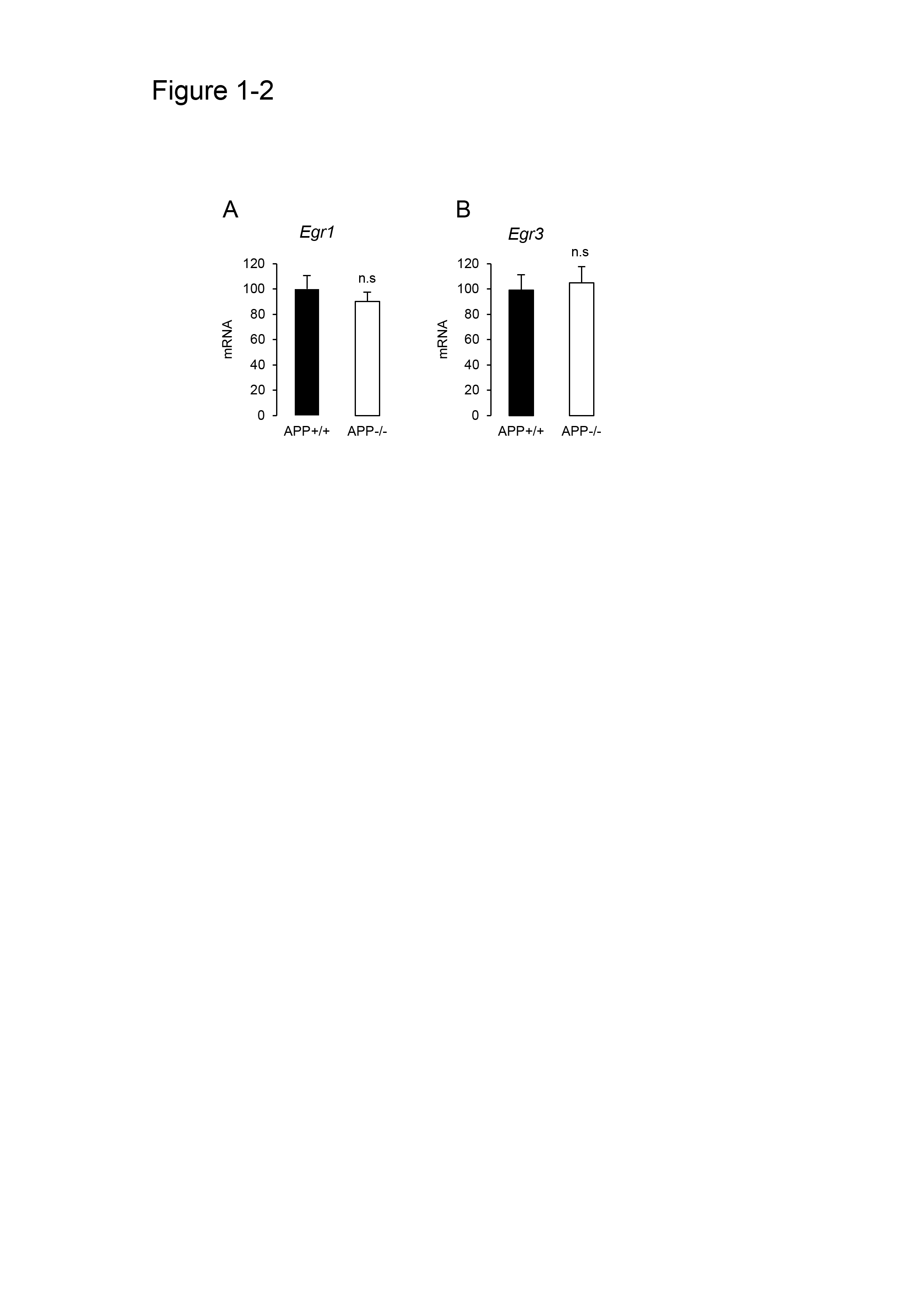

Supplement: Extended Data Figure 1-2 — Expression of Egr1 and Egr3 is not modified in APP−/− neurons. Egr1 and Egr3 expressions were evaluated in APP+/+ versus APP−/− primary neurons at DIV7. Egr1 mRNA (A) and Egr3 mRNA (B) levels was measured by qPCR (n = 6, N = 3) at DIV7. Results (mean ± SEM) are given as percentage of controls (APP+/+); n.s. = non-significant, Student’s t test. Download Figure 1-2, TIF file. [file enu-eN-NWR-0322-19-s03.tif]

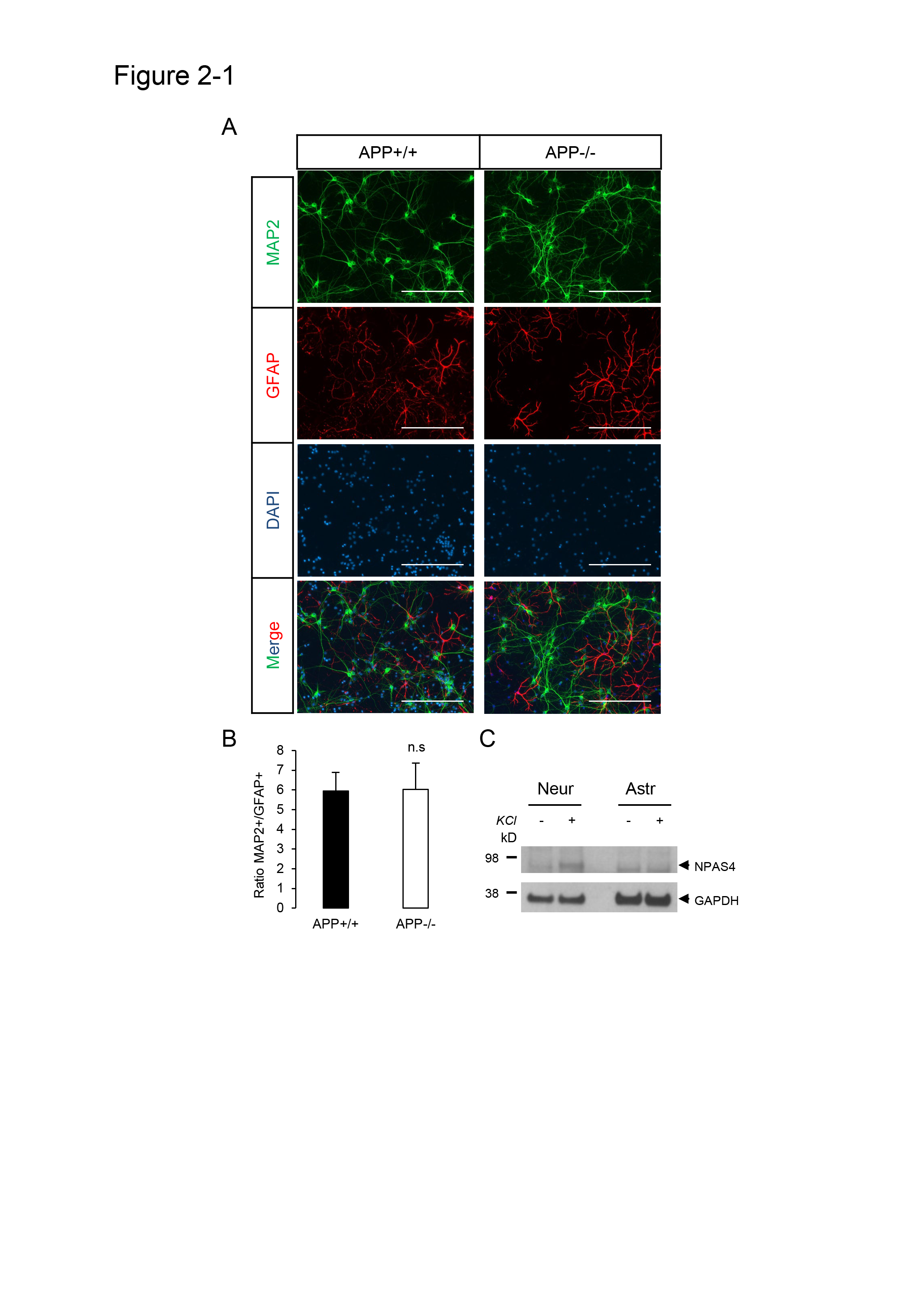

Supplement: Extended Data Figure 2-1 — Astrocytes in primary neuron culture and their implication in Npas4 expression. A, Primary culture of cortical neurons at DIV7. Cultures were immunostained with the neuron-specific protein MAP2 (green), the glial-specific protein GFAP (red) and the DAPI (light blue). Scale bar = 400 µm. B, Quantification of neurons (MAP2+) and astrocytes (GFAP+) in the primary cortical culture. At least five fields per coverslip were analyzed for APP+/+ and APP−/− cultures in two independent experiments (n ≥ 5, N = 2). Results are expressed as the ratio of MAP2+ (neurons) and GFAP+ (astrocytes; mean ± SEM); n.s. = non-significant, Mann–Whitney test. C, Western blot analysis of NPAS4 induction in neurons and astrocytes after depolarization with 50 mm KCl for 2 h. Download Figure 2-1, TIF file. [file enu-eN-NWR-0322-19-s04.tif]

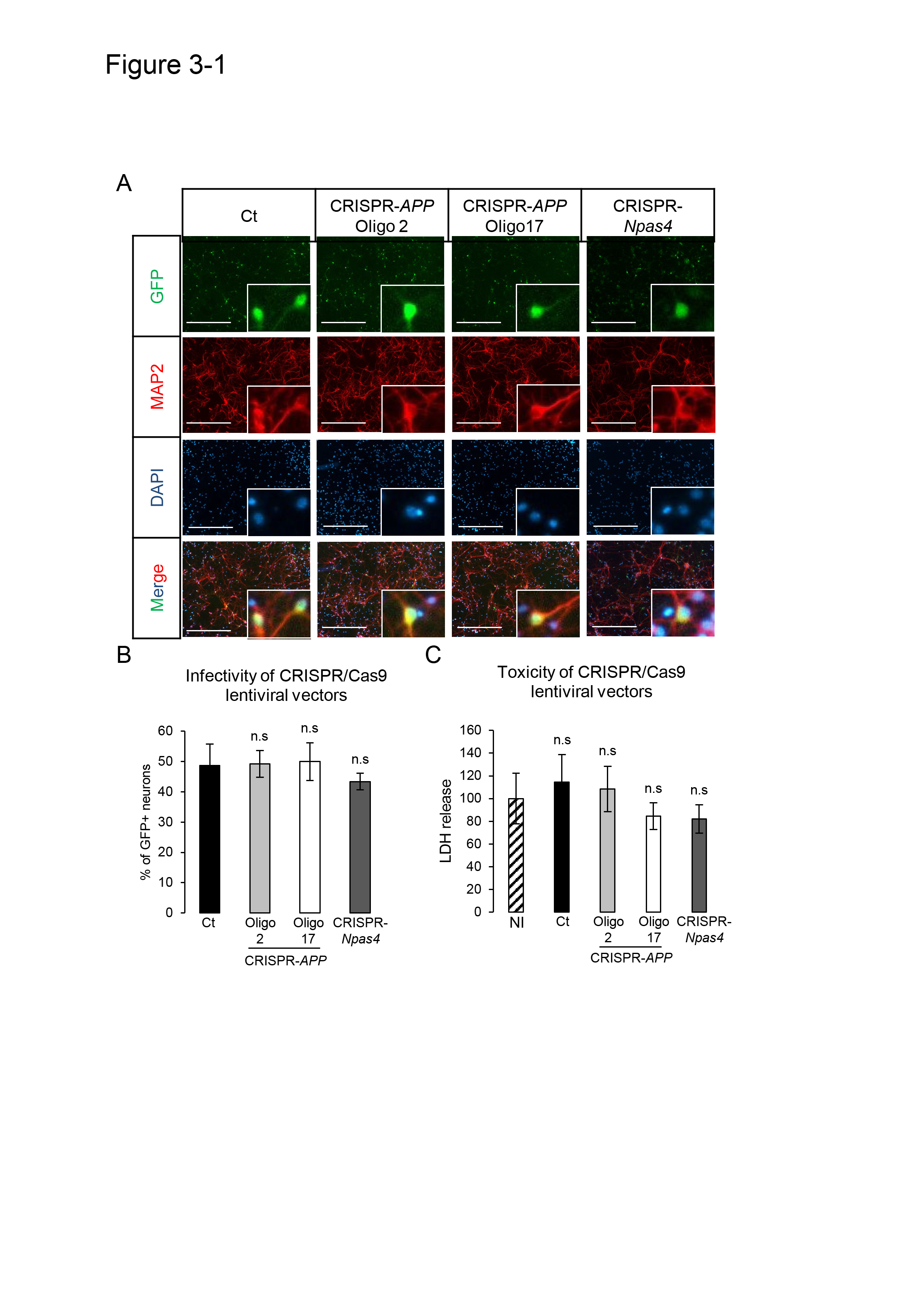

Supplement: Extended Data Figure 3-1 — Infectivity and toxicity of lentiviral CRISPR-Cas9 vectors. A, Cortical neurons were infected at DIV1 with lentiviruses expressing sgRNAs (Oligo2, Oligo17, or CRISPR-NPAS4) or no sgRNA (Ct), SpCas9, and GFP. Cultures were immunostained for MAP2 (red) and DAPI (light blue) at DIV7. Scale bar = 400 µm. B, Quantification of GFP+ neurons (GFP+/MAP2+) in total neuron population (MAP2+) after lentiviral CRISPR-Cas9 infection with control (Ct), Oligo2, Oligo17, or CRISPR-NPAS4. At least five fields were analyzed for each lentiviral vector in two independent experiments (n ≥ 5, N = 2). Results are expressed as percentage of GFP+/MAP2+ cells in total MAP2+ cells (mean ± SEM); n.s. = non-significant, Kruskal–Wallis test and Dunn’s multiple comparison test. C, Measurement of LDH activity released after infection (DIV7) of primary neuron with control (Ct), Oligo2, Oligo17, or CRISPR-NPAS4 at DIV7 lentiviral vectors. Background LDH release was determined in non-infected control cultures (NI). Results were expressed as percentage of total LDH release measured in non-infected control cultures (NI) in two independent experiments (n = 12, N = 2). Download Figure 3-1, TIF file. [file enu-eN-NWR-0322-19-s05.tif]

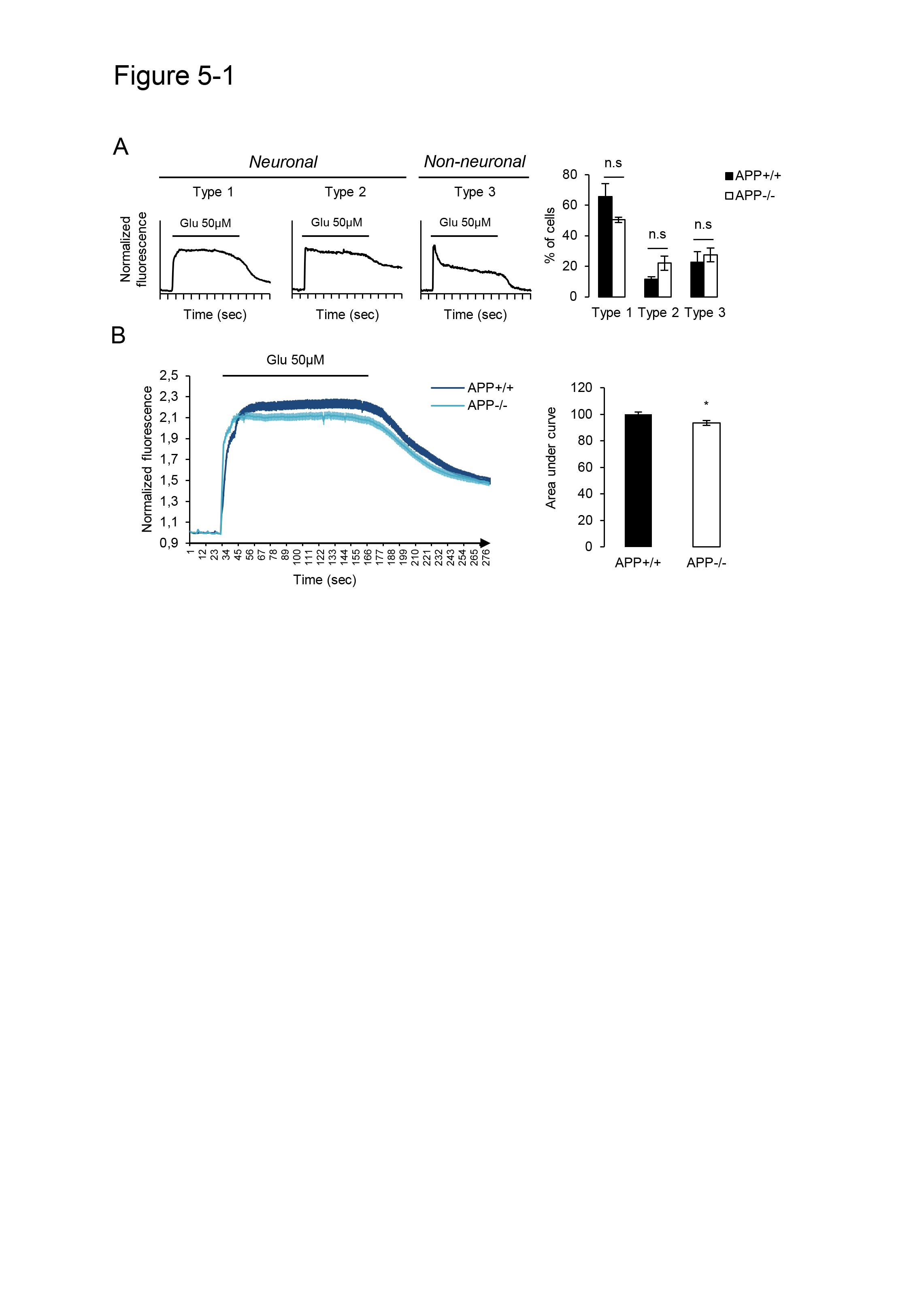

Supplement: Extended Data Figure 5-1 — Glutamate responses in APP−/− neurons measured by calcium imaging. Neuronal activity was measured at DIV7 by calcium imaging. A, left panel, Different calcium responses were observed after stimulation with 50 µm glutamate and classified as described by Pickering et al. (2008) between neuronal and non-neuronal responses. To note x-axis graduation corresponds to 20 s. Right panel, The proportion of cells displaying type 1, 2, or 3 response was quantified in three independent experiments (n = 9, N = 3); n.s. = non-significant. Student’s t test. B, Normalized fluorescence trace (mean ± SEM) measured in APP+/+ and APP−/− neurons upon perfusion for 150 s with 50 µm glutamate. The AUC was quantified for 50 neurons per coverslips. A total of nine coverslips for each genotype was recorded in three independent experiments (N = 3). The graph on the right shows AUC expressed as percentage of control (APP+/+); *p = 0.0106, Student’s t test. Download Figure 5-1, TIF file. [file enu-eN-NWR-0322-19-s06.tif]

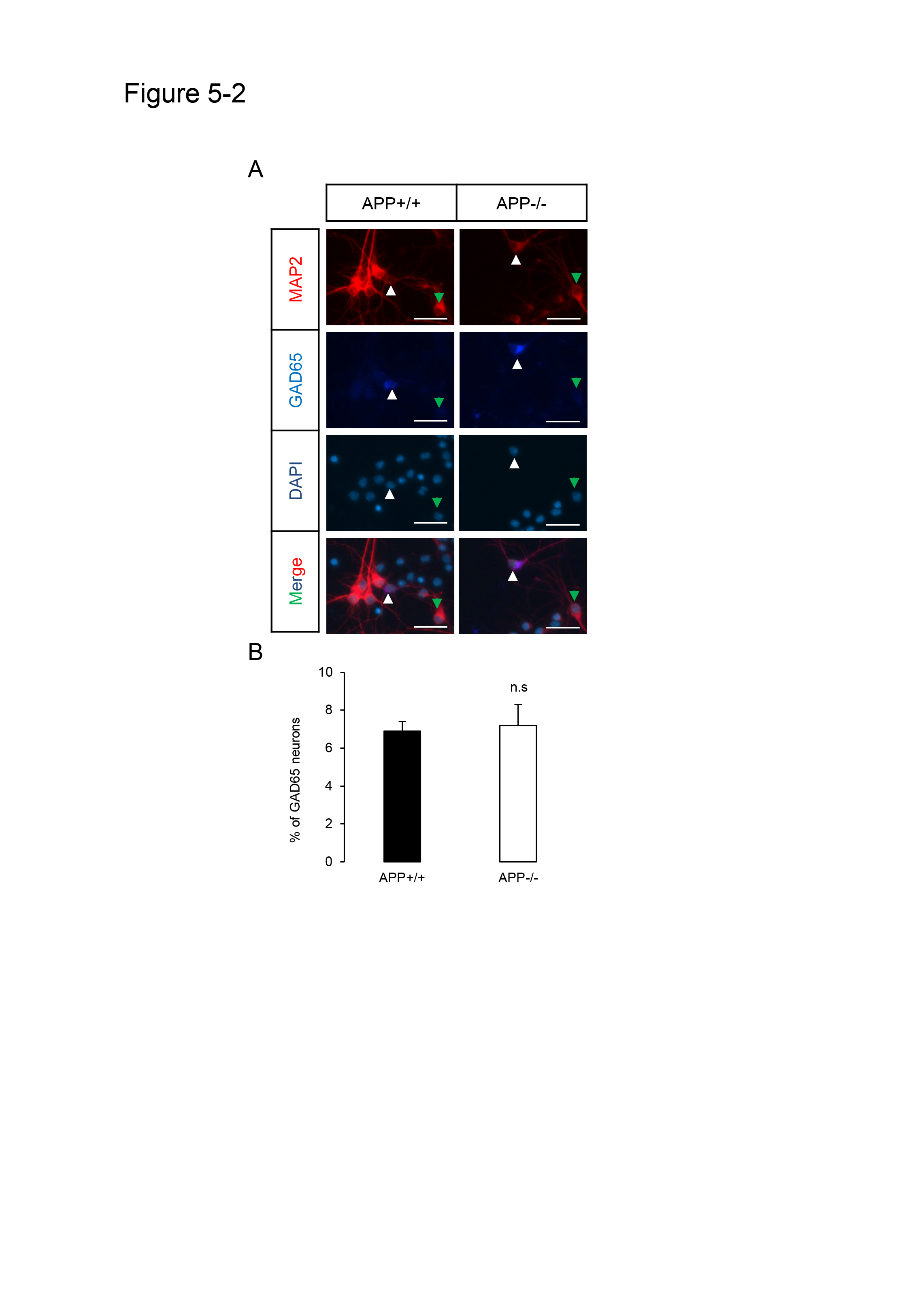

Supplement: Extended Data Figure 5-2 — GAD65-positive neurons in primary cortical cultures. A, Primary culture of cortical neurons after at DIV7. Cultures were immunostained with the neuron-specific protein MAP2 (red), GAD65 (dark blue), and DAPI (light blue). Representative 20× micrographs show GAD65-positive neurons (white arrowhead) and GAD65-negative neuron (green arrowhead). B, Images (20× objective) were quantified (10 fields per coverslip for each genotype) in three independent cultures (n = 30, N = 3). Results (mean ± SEM) are expressed as percentage of GAD65+ MAP2+ cells (GAD65+ neurons) among all MAP2+ cells (neurons); n.s. = non-significant, Mann–Whitney test. Scale bar = 20 µm. Download Figure 5-2, TIF file. [file enu-eN-NWR-0322-19-s07.tif]

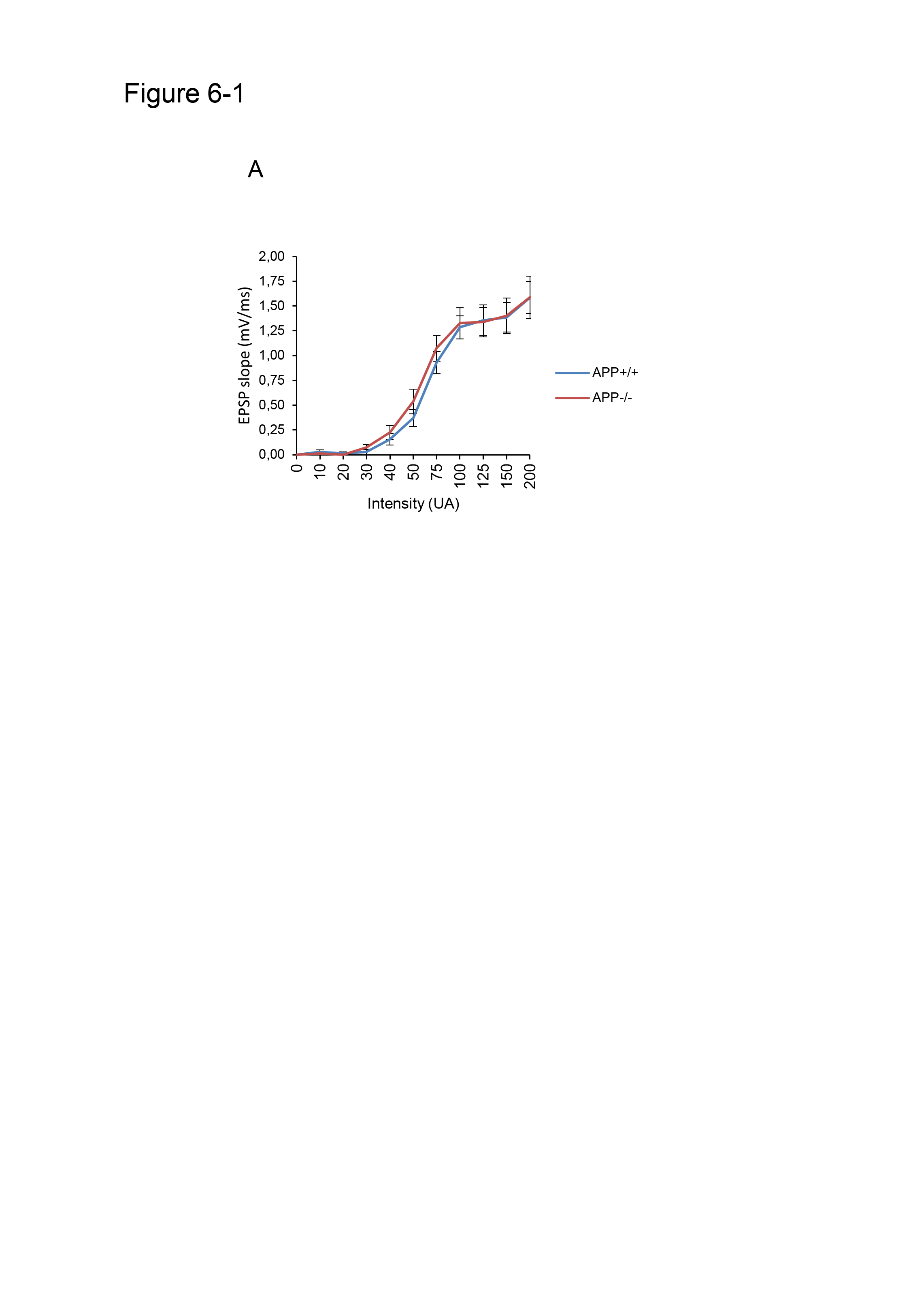

Supplement: Extended Data Figure 6-1 — LTP in hippocampal SC-CA1 pathway in APP−/− mice. Excitatory postsynaptic potentials measured in hippocampal CA1 region of brain slices from APP+/+ (N = 9) and APP−/− mice (N = 8). A, The input-output relationship between fEPSP measured in CA1 stratum radiatum and the intensity of SC stimulation is represented. No significant difference between APP+/+ and APP−/− was observed. Download Figure 6-1, TIF file. [file enu-eN-NWR-0322-19-s08.tif]
